# Supplementary material for: Cheminformatics and Machine Learning Approaches to Assess Aquatic Toxicity Profiles of Fullerene Derivatives
Source: Int J Mol Sci. 2023 Sep 15;24(18):14160. doi: 10.3390/ijms241814160 (PMC10531479; doi:10.3390/ijms241814160)
Supplement: Supplementary file 1 [file ijms-24-14160-s001.zip › Supplementary Materials S1 (SMS1).pdf]

Cheminformatics and Machine Learning Approaches to Assess Aquatic Toxicity Profile of Fullerene Derivatives

Natalja Fjodorova [1,*], Marjana Novič [1], Katja Venko [1], Bakhtiyor Rasulev [2], Melek Türker Saçan [3], Gulcin Tugcu [4], Safiye Sağ Erdem [5], Alla P. Toropova [6] and Andrey A. Toropov [6]

Supplementary Materials S1 (SMS1)

Table of content:

Table S1. The statistical performance of CPANN model (*ALL_CPANN model*) based on twenty eight descriptors (twenty five drug-like descriptors, QPpolrz, TD and DCW).
Table S2. Summary matrix with the ranges of correlation coefficients which describe relationships between binding score of ten proteins related to aquatic species, average BScores, and binding affinity.
Table S3. Summary matrix with the ranges of correlation coefficients which describe relationships between binding scores of ten proteins related to aquatic species and descriptors.
Figure S1. Plots for comparison binding scores for 10 proteins associated with aquatic toxicity with Average Binding Scores.
Figure S2. Plot for comparison binding scores for protein 1VOT related to *Tc*AChE with Average Binding Scores.
Table S4. Regression equations and performance of regression models (*ALL_regression model*) using twenty eight descriptors (twenty five drug like descriptors, QPpolrz, TD and DCW).
Table S5. Regression equations and performance of regression models using two descriptors: polarizability volume in cubic angstroms (QPpolrz) and topological descriptor (TD) related to Model 1_regression.
Table S6. Regression equations and performance of regression models using Monte Carlo optimal descriptor DCW related to Model 2_regression.
Figure S3. The plots of actual response (binding affinity) vs. predicted one for Model _regression: for the following responses: (a)-Average BScores (R^2=0.93); (b)-binding affinity (R^2=0.90); binding scores for (c)- 1D6U (R^2=0.34); (d)- 1E3K (R^2=0.88); (e)- 1GOS (R^2=0.93); (f)- 1GS4 (R^2=0.81); (g)- 1H82 (R^2=0.84); (h)- 1OG5 (R^2=0.84); (i)- 1UOM (R^2=0.85); (j)- 2F9Q (R^2=0.86); (k)- 2J0D (R^2=0.83); (l)- 3ERT (R^2=0.65).
Figure S4. The plots of actual response (binding affinity) vs. predicted one for Model 2_regression: (a)-Average BScores (R^2=0.93); (b)-binding affinity (R^2=0.91); binding scores for (c)- 1D6U (R^2=0.38); (d)- 1E3K (R^2=0.86); (e)- 1GOS (R^2=0.87); (f)- 1GS4 (R^2=0.80); (g)- 1H82 (R^2=0.85); (h)- 1OG5 (R^2=0.85); (i)- 1UOM (R^2=0.81); (j)- 2F9Q (R^2=0.84); (k)- 2J0D (R^2=0.84); (l)- 3ERT (R^2=0.63).
Figure S5. Williams plots: standardized residuals versus leverage for Model 1_regression based on QPpolrz and topological diameter (TD) for the following responses: (a)-Average BScore; (b)-binding affinity; binding scores for (c)- 1D6U; (d)- 1E3K; (e)- 1GOS; (f)- 1GS4.
Figure S6. Williams plots: standardized residuals versus leverage for Model 1_regression based on QPpolrz and topological diameter (TD) for the following responses: binding scores for (g)- 1H82; (h)- 1OG5; (i)- 1UOM; (j)- 2F9Q; (k)- 2J0D; (l)- 3ERT.
Table S7. FDs outside the limits: warning leverage threshold (h*) and outside the square area between ±3 standard deviation units (σ) in *Model 1_regression* related to responses: (a)-Average BScores; (b)-Binding affinity; binding scores for :(c)- 1D6U; (d)- 1E3K; (e)- 1GOS; (f)- 1GS4; (g)- 1H82; (h)- 1OG5; (i)- 1UOM; (j)- 2F9Q; (k)- 2J0D; (l)- 3ERT.

Table S8. Structure of chemicals (FDs) outside the limits: warning leverage threshold (h*) and outside the square area between ±3 standard deviation units (σ) in *Model 1_regression*.

Figure S7. Williams plots: standardized residuals versus leverage for Model 2_regression based on the optimal Monte Carlo descriptors for the following responses: (a)-Average BScore; (b)-binding affinity; binding scores for (c)- 1D6U; (d)- 1E3K; (e)- 1GOS; (f)- 1GS4.

Figure S8. Williams plots: standardized residuals versus leverage for Model 2_regression based on the optimal Monte Carlo descriptors for the following responses: binding scores for: (g)- 1H82; (h)- 1OG5; (i)- 1UOM; (j)- 2F9Q; (k)- 2J0D; (l)- 3ERT.

Table S9. FDs outside the limits: warning leverage threshold (h*) and outside the square area between ±3 standard deviation units (σ) in *Model 2_regression* related to responses: (a)-Average BScores; (b)-binding affinity; binding scores for: (c)- 1D6U; (d)- 1E3K; (e)- 1GOS; (f)- 1GS4; (g)- 1H82; (h)- 1OG5; (i)- 1UOM; (j)- 2F9Q; (k)- 2J0D; (l)- 3ERT.

Table S10. Structure of chemicals (FDs) outside the limits: warning leverage threshold (h*) and outside the square area between ±3 standard deviation units (σ) in *Model 2_regression*.

Table S11. The statistical performance of *Model 1_CPANN* (M1) and *CPANN model 2*(M2) related to the training set.

Table S12. The statistical performance of *CPANN model 1*(M1) and *CPANN model 2*(M2) related to the test set.

Table S13. The statistical performance of *CPANN model 1*(M1) and *CPANN model 2*(M2) related to validation leave one out (LOO) procedure.

Figure S9. The plots of target response (binding affinity) vs. predicted one for *Model 1_CPANN*: for the following responses: (a)-Average BScore; (b)-binding affinity; binding scores for (c)- 1D6U; (d)- 1E3K; (e)- 1GOS; (f)- 1GS4; (g)- 1H82; (h)- 1OG5; (i)- 1UOM; (j)- 2F9Q; (k)- 2J0D; (l)- 3ERT.

Figure S10. The plots of target response (binding affinity) vs. predicted one for *Model 2_CPANN*: for the following responses: (a)-Average BScore; (b)-binding affinity; binding scores for (c)- 1D6U; (d)- 1E3K; (e)- 1GOS; (f)- 1GS4; (g)- 1H82; (h)- 1OG5; (i)- 1UOM; (j)- 2F9Q; (k)- 2J0D; (l)- 3ERT.

Table S1. The statistical performance of CPANN model (*ALL_CPANN model*) based on twenty eight descriptors (twenty five drug-like descriptors, QPpolrz, TD and DCW).

**Response (Binding affinity)**	Squared Correlation coefficient (R^2)	Root Mean Squared Error (RMSE)
(1) Average Binding Scores	0.995	0.070
(2) Binding affinity	0.995	0.068
(3) Binding Scores for 1D6U	0.988	0.111
(4) Binding Scores for 1E3K	0.992	0.090
(5) Binding Scores for 1GOS	0.985	0.124
(6) Binding Scores for 1GS4	0.984	0.128
(7) Binding Scores for 1H82	0.987	0.114
(8) Binding Scores for 1OG5	0.980	0.143
(9) Binding Scores for 1UOM	0.986	0.119
(10) Binding Scores for 2F9Q	0.992	0.091
(11) Binding Scores for 2J0D	0.991	0.094
(12) Binding Scores for 3ERT	0.965	0.188

Table S2. Summary matrix with the ranges of correlation coefficients which describe relationships between binding score of ten proteins related to aquatic species, average BScores, and binding affinity. Calculation was done using the Minitab statistical program.

**Output variables- binding activities**	**The range of correlation coefficients**
Average BScores (Av.Bsc.)<br>*Binding affinity* (Bind.af.)	0.966 (Av. Bsc. vs.1E3K) - 0.656 (Av. Bsc.vs. 1D6U)<br>0.968 (Bind. af.vs. 1E3K) - 0.669 (Bind. af.vs. 1D6U)
Binding scores of ten proteins related to aquatic species: 1D6U, 1E3K, 1GOS, 1GS4, 1H82, 1OG5, 1UOM, 2F9Q, 2J0D, 3ERT	0.937 (2J0D vs. 2F9Q) - 0.570 (1D6U vs.1E3K)

Table S3. Summary matrix with the ranges of correlation coefficients which describe relationships between binding scores of ten proteins related to aquatic species and descriptors.

**Input variables-descriptors**	**The range of correlation coefficients**
Non H-atoms(NH-a)	0.863 (NH-a vs. 2JOD) - 0.494 (NH-a vs. 1D6U)
Rotatable Bonds (RB)	0.797 (RB vs. 2F9Q) - 0.517 (RB vs. 3ERT)
Molecular weight (MW)	0.824 (MW vs. 2JOD) - 0.460 (MW vs. 1D6U)
Total Surface Area (TSA)	0.904 (TSA vs. 2JOD) - 0.569 (TSA vs. 1D6U)
Topological Diameter (TD)	0.890 (TD vs.1UOM) - 0.443 (TD vs. 1D6U)
QPpolrz	0.937 (QPpolrz vs. 2F9Q)-0.614 (QPpolrz vs.1D6U)
DCW	0.932 (DCW vs. 1GOS) - 0.601 (DCW vs.1D6U)

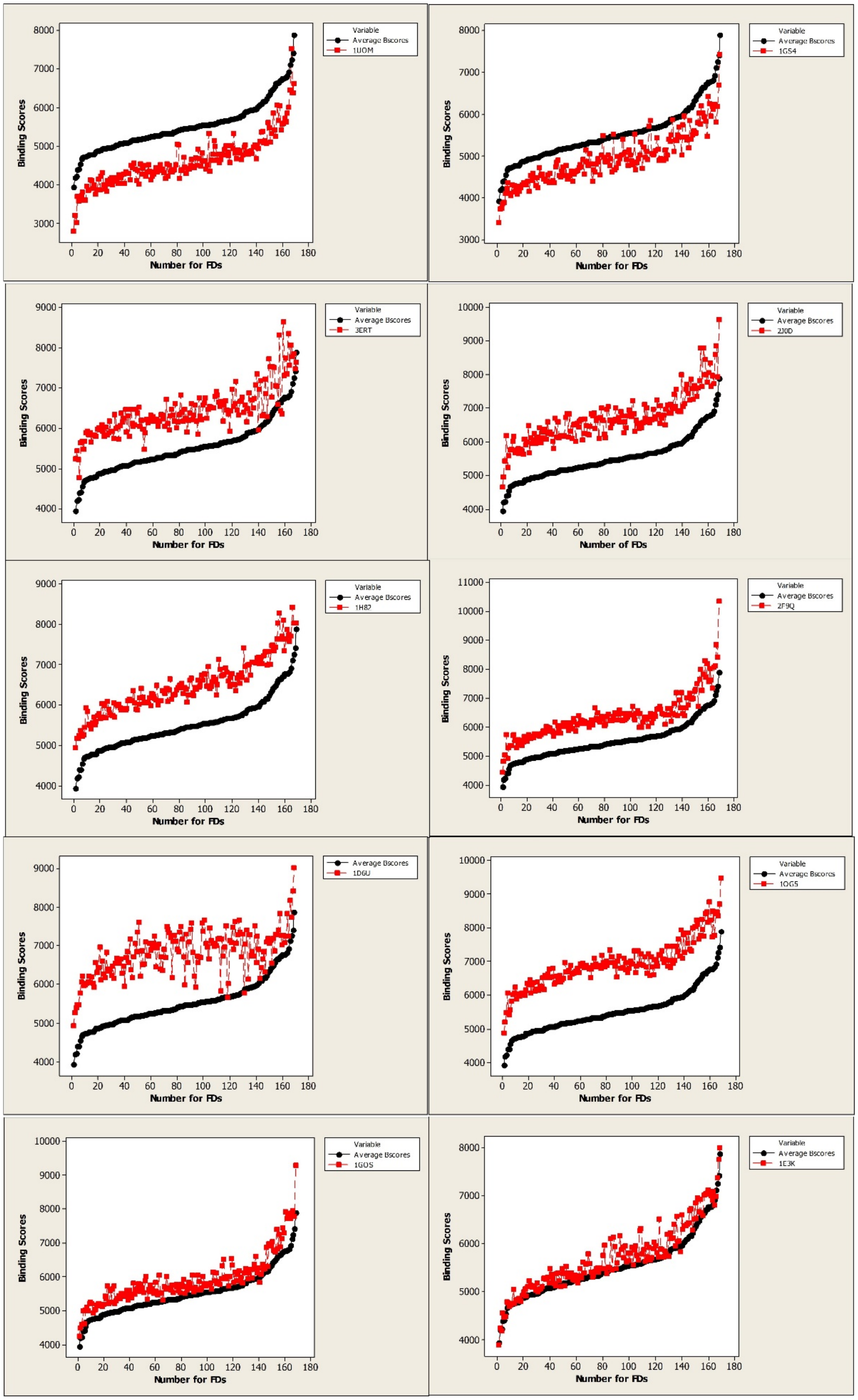

Figure S1. Plots for comparison binding scores for 10 proteins associated with aquatic toxicity with Average Binding Scores.

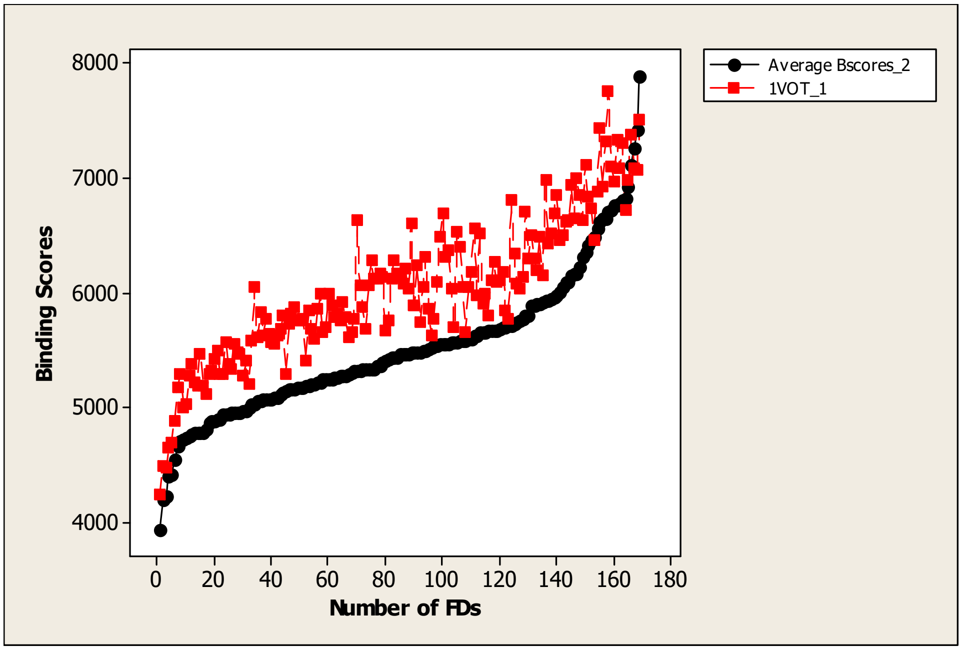

Figure S2. Plot for comparison binding scores for protein 1VOT related to *Tc*AChE with Average Binding Scores.

Table S4. Regression equations and performance of regression models (*ALL_regression model*) using twenty eight descriptors (twenty five drug like descriptors, QPpolrz, TD and DCW).

**Response (Binding affinity)**	**Regression equation**	**R^2(adj)**
**Average BScores**	**Average Bscores** = 2464 + 17.6 DCW + 20.1 QPpolrz + 56.1 TD – 58.6 H-Acceptors+ 3.47 Total Surface Area + 1570 Relative PSA- 8.88 Molweight+ 33.3 cLogP + 60.4 Electronegative Atoms- 9.56 Stereo Centers- 79.0 Rings Closures + 35.3 Small Rings - 134 Aromatic Rings+ 30.6 Aromatic Atoms + 17.1 sp3-Atoms + 95.2 Non-H Atoms	0.968
**Binding affinity**	**Binding affinity** = 3.49 + 0.00169 DCW + 0.00122 QPpolrz + 0.00463 TD- 0.00557 H-Acceptors + 0.183 Relative PSA+ 0.000040 Polar Surface Area – 0.000130 Druglikeness- 0.000626 Molweight + 0.00216 cLogP – 0.000983 Stereo Centers- 0.00815 Rings Closures+ 0.00308 Small Rings – 0.0130 Aromatic Rings+ 0.00271 Aromatic Atoms + 0.00146 sp3-Atoms + 0.00776 Non-H Atoms	0.952
**Binding scores related to proteins:**		
1D6U	**1D6U** = 9804 – 78.8 TD - 139 H-Acceptors+ 14.1 Total Surface Area + 4260 Relative PSA- 40.3 Molweight+ 166 Electronegative Atoms- 43.3 Stereo Centers - 274 Rings Closures- 627 Aromatic Rings + 134 Aromatic Atoms+ 71.6 sp3-Atoms – 6.42 Symmetric atoms + 494 Non-H Atoms	0.646
1E3K	**1E3K** = 1944 + 25.8 DCW + 26.4 QPpolrz + 59.9 TD + 7.63 Total Surface Area – 10.8 Molweight+ 98.6 Electronegative Atoms+ 58.0 Small Rings - 222 Aromatic Nitrogens	0.912
1GOS	**1GOS** = 2756 + 60.8 TD- 10.1 Druglikeness – 15.5 Molweight+ 38.5 cLogP + 90.1 Electronegative Atoms+ 46.0 Small Rings + 40.5 Aromatic Atoms+ 28.6 sp3-Atoms + 52.7 Amides + 182 Non-H Atoms	0.924
1GS4	**1GS4** = 1314 + 30.8 DCW + 75.0 TD + 1967 Relative PSA+ 57.6 cLogP - 185 Aromatic Nitrogens	0.846
1H82	**1H82** = 3064 + 25.8 DCW + 43.9 QPpolrz – 17.8 Stereo Centers + 50.6 Small Rings - 209 Aromatic Rings + 41.4 Aromatic Atoms	0.904
1OG5	**1OG5** = 3148 + 72.9 TD – 97.4 H-Acceptors+ 2834 Relative PSA- 14.9 Molweight – 23.5 Stereo Centers - 185 Rings Closures + 56.8 Small Rings + 37.3 sp3-Atoms + 260 Non-H Atoms	0.898

1UOM	**1UOM** = 1805 + 36.0 QPpolrz + 82.9 TD + 1675 Relative PSA- 14.9 Molweight+ 128 Electronegative Atoms+ 27.9 Rotatable Bonds - 208 Aromatic Nitrogens	0.876
2F9Q	**2F9Q** = 2130 + 49.4 TD - 121 H-Acceptors+ 2066 Relative PSA- 12.5 Molweight+ 40.1 cLogS – 15.1 Stereo Centers + 29.9 sp3-Atoms + 173 Non-H Atoms	0.933
2J0D	**2J0D** = 2043 – 79.4 H-Acceptors – 27.0 H-Donors + 8.74 Total Surface Area – 26.9 Stereo Centers + 26.2 sp3-Atoms	0.934
3ERT	**3ERT** = 6769 + 36.5 QPpolrz + 2233 Relative PSA- 11.3 Druglikeness – 29.0 Molweight+ 79.8 cLogP + 205 Electronegative Atoms- 292 Aromatic Rings + 71.8 Aromatic Atoms + 46.3 sp3-Atoms + 262 Non-H Atoms	0.812

*** For more visual clarity we highlighted in Table S4 the same descriptors in different equations with the same color.

Discussion about features affecting the binding affinity using regression equations

Descriptors express the features transformed from the structure of FDs. Therefore, it is important to determine descriptors that affect the binding affinity the most. Such research will contribute to the mechanistic interpretation of our models and understanding of the influence of the most significant factors affecting the protein-ligand binding affinity. Regression analysis was implemented to find out relationships between descriptors and average BScores and binding affinity as well as relationships between descriptors and binding scores related to ten proteins associated with aquatic toxicity.

As a result, we have got regression equations for the responses: average BScores, binding affinity, and binding scores for the ten proteins. See Table S4. All descriptors (twenty-five drug-like descriptors, QPpolrz, TD and DCW) have been used in regression models represented in this Table S4.

The significant contribution to the average BScore activity belongs to the following descriptors: DCW, QPpolrz, topological diameter (TD), H-Acceptors, Total Surface Area, Relative PSA, Molweight, cLogP, Electronegative Atoms, Stereo Centers, Rings Closures, Small Rings, Aromatic Rings, Aromatic Atoms, sp^3-Atoms, and Non-H Atoms.

Considering the equations related to binding scores of ten proteins associated with aquatic toxicity (Table S4) we can make the following conclusion. The largest contributor in all cases is the topological diameter (TD). The size of FDs is significant for all responses except 1H82, 2J0D and 3ERT. Then follow QPpolrz and DCW. The Molecular weight and Non-H Atoms appeared to be important in the majority of cases too with the exception of 1GS4, 1H82, and a few others.

Table S5. Regression equations and performance of regression models using two descriptors: polarizability volume in cubic angstroms (QPpolrz) and topological descriptor (TD) related to Model 1_regression.

Response (Binding affinity)	Regression equation	R^2(adj)
**Average BScores**	Average Bscores = 1516 + 42.5 QPpolrz + 73.0 TD	0.934
**Binding affinity**	Binding affinity = 3.44 + 0.00308 QPpolrz + 0.00590 TD	0.906
**Binding scores related to proteins:**		
1D6U	1D6U = 4110 + 47.5 QPpolrz - 39,2 TD	0.366
1E3K	1E3K = 1480 + 42.1 QPpolrz + 89,7 TD	0.877
1GOS	1GOS = 1398 + 46.4 QPpolrz + 89,2 TD	0.893
1GS4	1GS4 = 1298 + 38.3 QPpolrz + 68,5 TD	0.812
1H82	1H82 = 2349 + 44.7 QPpolrz + 71,3 TD	0.846
1OG5	1OG5 = 2590 + 49.9 QPpolrz + 61,4 TD	0.848
1UOM	1UOM = 896 + 30.3 QPpolrz + 110 TD	0.850
2F9Q	2F9Q = 1425 + 63.9 QPpolrz + 35,5 TD	0.882
2J0D	2J0D = 1850 + 67.0 QPpolrz + 16,3 TD	0.853
3ERT	3ERT = 3726 + 12.0 QPpolrz + 126 TD	0.660

Table S6. Regression equations and performance of regression models using Monte Carlo optimal descriptor DCW related to Model 2_regression.

Response (Binding affinity)	Regression equation	R^2(adj)
**Average Bscores**	Average Bscores = 309 + 76.8 DCW	0.926
**Binding affinity**	Binding affinity = 3.34 + 0.00583 DCW	0.912
**Binding scores related to proteins:**		
1D6U	1D6U = 3653 + 46.3 DCW	0.373
1E3K	1E3K = 148 + 81.9 DCW	0.860
1GOS	1GOS = 53 + 86.3 DCW	0.867
1GS4	1GS4 = 203 + 69.9 DCW	0.800
1H82	1H82 = 1090 + 79.5 DCW	0.853
1OG5	1OG5 = 1388 + 81.7 DCW	0.844
1UOM	1UOM = - 434 + 74.3 DCW	0.808
2F9Q	2F9Q = 395 + 87.6 DCW	0.840
2J0D	2J0D = 819 + 86.6 DCW	0.843
3ERT	3ERT = 2361 + 59.8 DCW	0.624

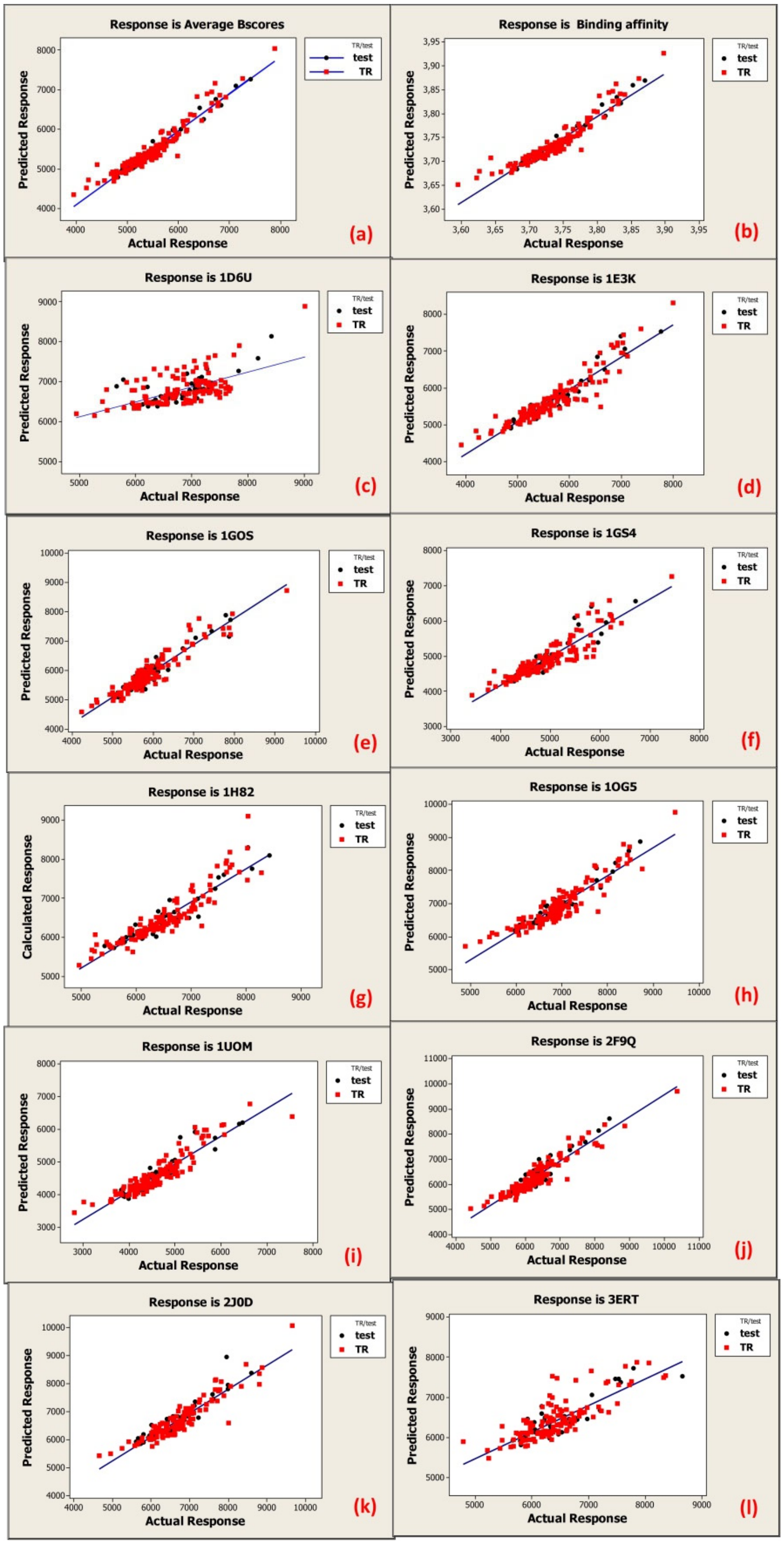

Figure S3. The plots of actual response (binding affinity) vs. predicted one for Model _regression: for the following responses: (a)-Average BScores (R^2=0.93); (b)-binding affinity (R^2=0.90); binding scores for (c)-1D6U (R^2=0.34); (d)- 1E3K (R^2=0.88); (e)- 1GOS (R^2=0.93); (f)- 1GS4 (R^2=0.81); (g)- 1H82 (R^2=0.84); (h)-1OG5 (R^2=0.84); (i)- 1UOM (R^2=0.85); (j)- 2F9Q (R^2=0.86); (k)- 2J0D (R^2=0.83); (l)- 3ERT (R^2=0.65).

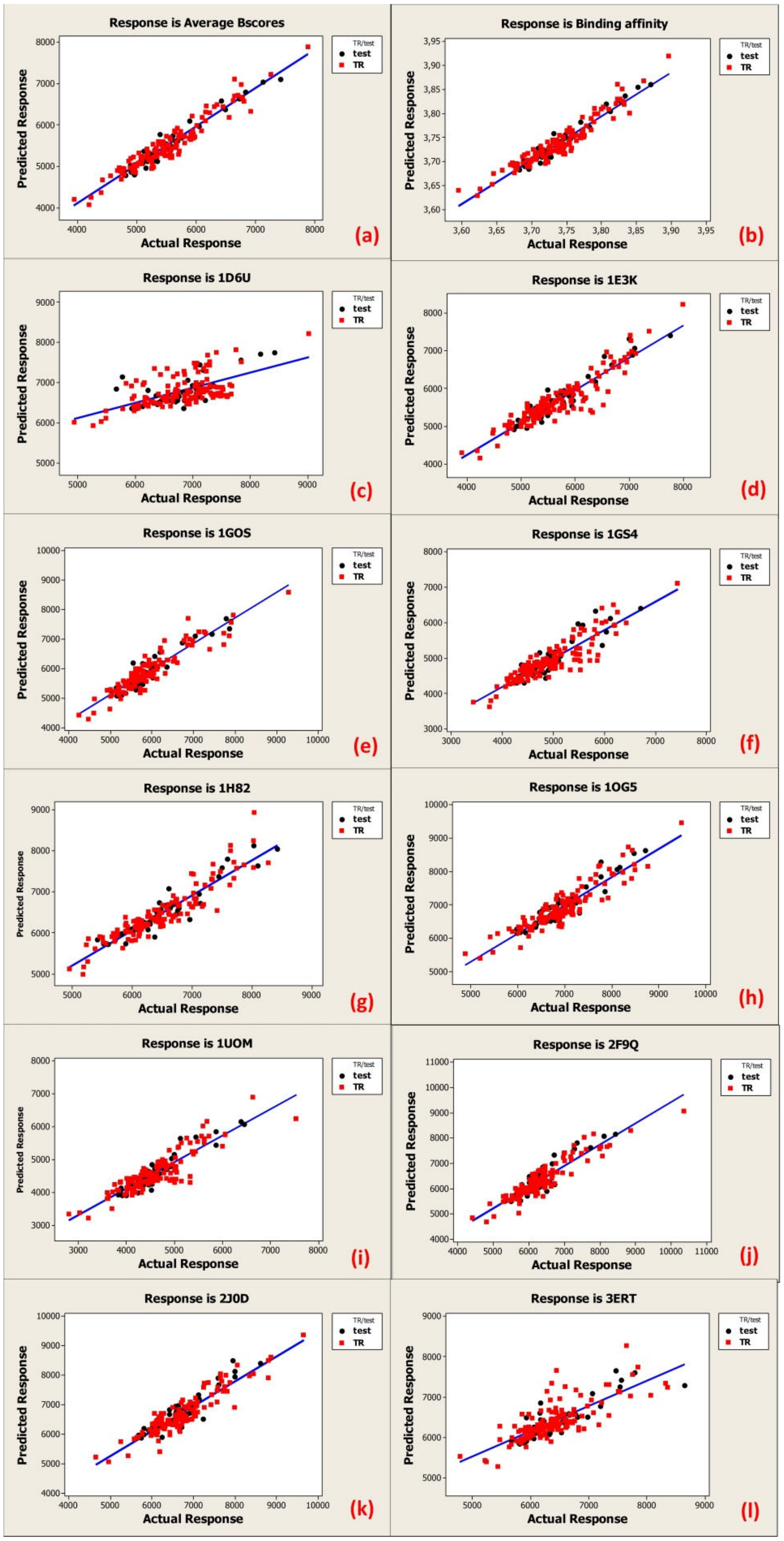

Figure S4. The plots of actual response (binding affinity) vs. predicted one for Model 2_regression: (a)-Average BScores (R^2=0.93); (b)-binding affinity (R^2=0.91); binding scores for (c)- 1D6U (R^2=0.38); (d)- 1E3K (R^2=0.86); (e)- 1GOS (R^2=0.87); (f)- 1GS4 (R^2=0.80); (g)- 1H82 (R^2=0.85); (h)- 1OG5 (R^2=0.85); (i)- 1UOM (R^2=0.81); (j)- 2F9Q (R^2=0.84); (k)- 2J0D (R^2=0.84); (l)- 3ERT (R^2=0.63).

Applicability domain of QSAR Models

Model 1.

Williams plots were employed for the visualization applicability domain (AD) of QSAR models. The leverage values (or hat values) were plotted against the standardized residuals for each FD. For details about AD and plots used in this method see the paper written by Gramatica, (2007). The goal of the Williams plot is to demonstrate a relationship between the leverage values (expressing the similarity of a given compound to the training set) and the standardized residuals (prediction errors observed for specific compounds).

Figures S5 and S6 represents the Williams plots for Model 1_regression based on two descriptors (QPpolrz and TD) while Figures S7 and S8 demonstrates the Williams plots in the case of Model 2_regression based on optimal DCW descriptor. The plots were built for all output variables: Average BScores (1), Binding affinity (2), and Binding scores for ten proteins.

The warning leverage (h^*) is generally set to $3(p+1)/n$, where n is the number of training chemicals and p is the number of model variables (descriptors) plus one. In the case of Model 1_regression, the h^* value was set to 0.7, while in Model 2_regression, the h^* value was set to 0.47.

We have examined chemicals characterized by leverage (hat) value that exceeds the threshold for the warning leverage because they are influential in the structural domain of the model. The chemicals that fall outside ±3 standard deviation units were considered also as they indicate the prediction errors.

Figure S5 demonstrated Williams plots for the following output variables: (a)-Average BScore; (b)-Binding affinity; Binding scores for (c)- 1D6U; (d)- 1E3K; (e)- 1GOS; (f)- 1GS4, while Figure S6 illustrated Williams plots for output variables: Binding scores for (g)- 1H82; (h)- 1OG5; (i)- 1UOM; (j)- 2F9Q; (k)- 2J0D; (l)- 3ERT.

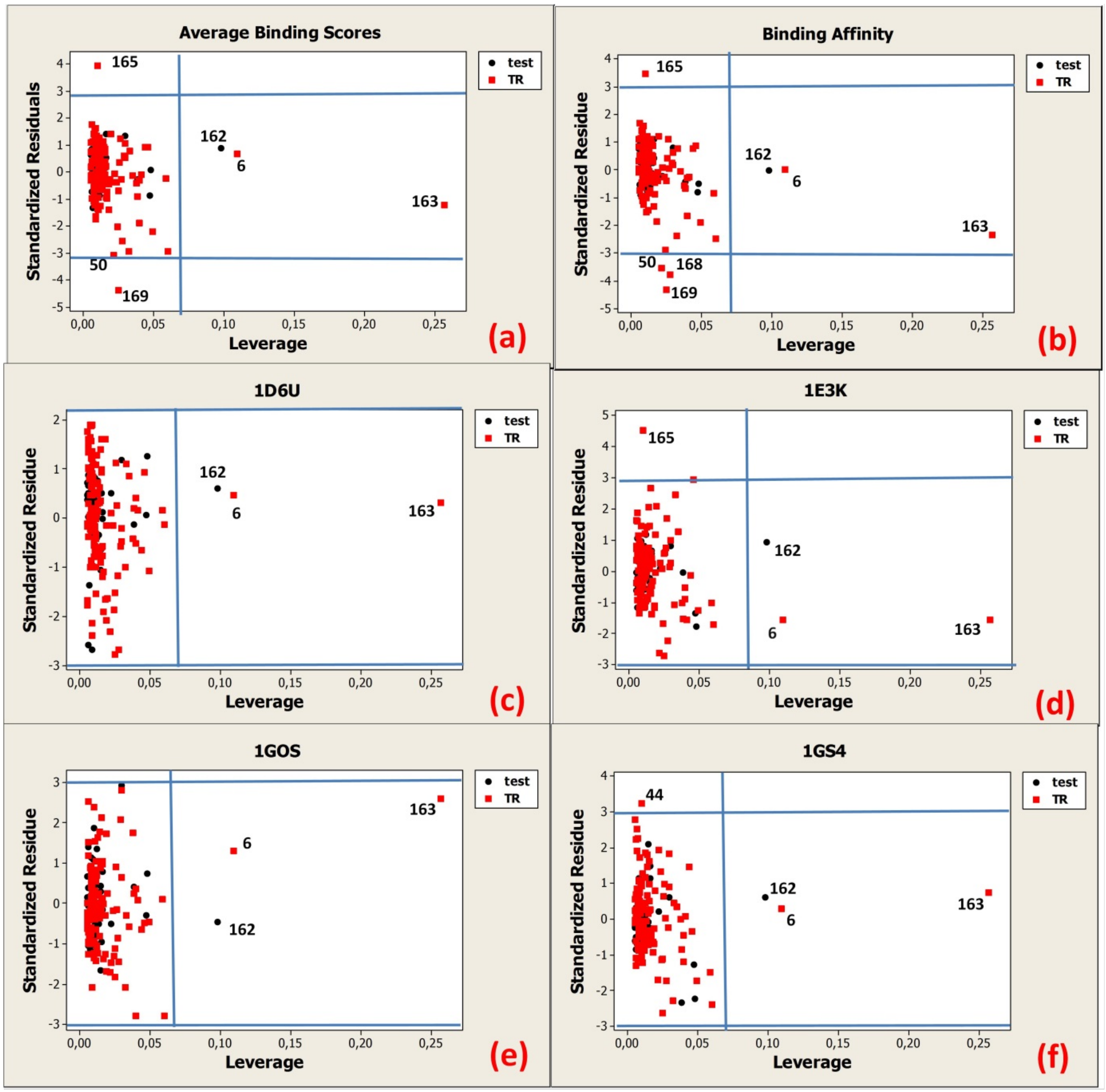

Figure S5. Williams plots: standardized residuals versus leverage for Model 1_regression based on QPpolrz and topological diameter (TD) for the following responses: (a)-Average BScore; (b)-binding affinity; binding scores for (c)- 1D6U; (d)- 1E3K; (e)- 1GOS; (f)- 1GS4.

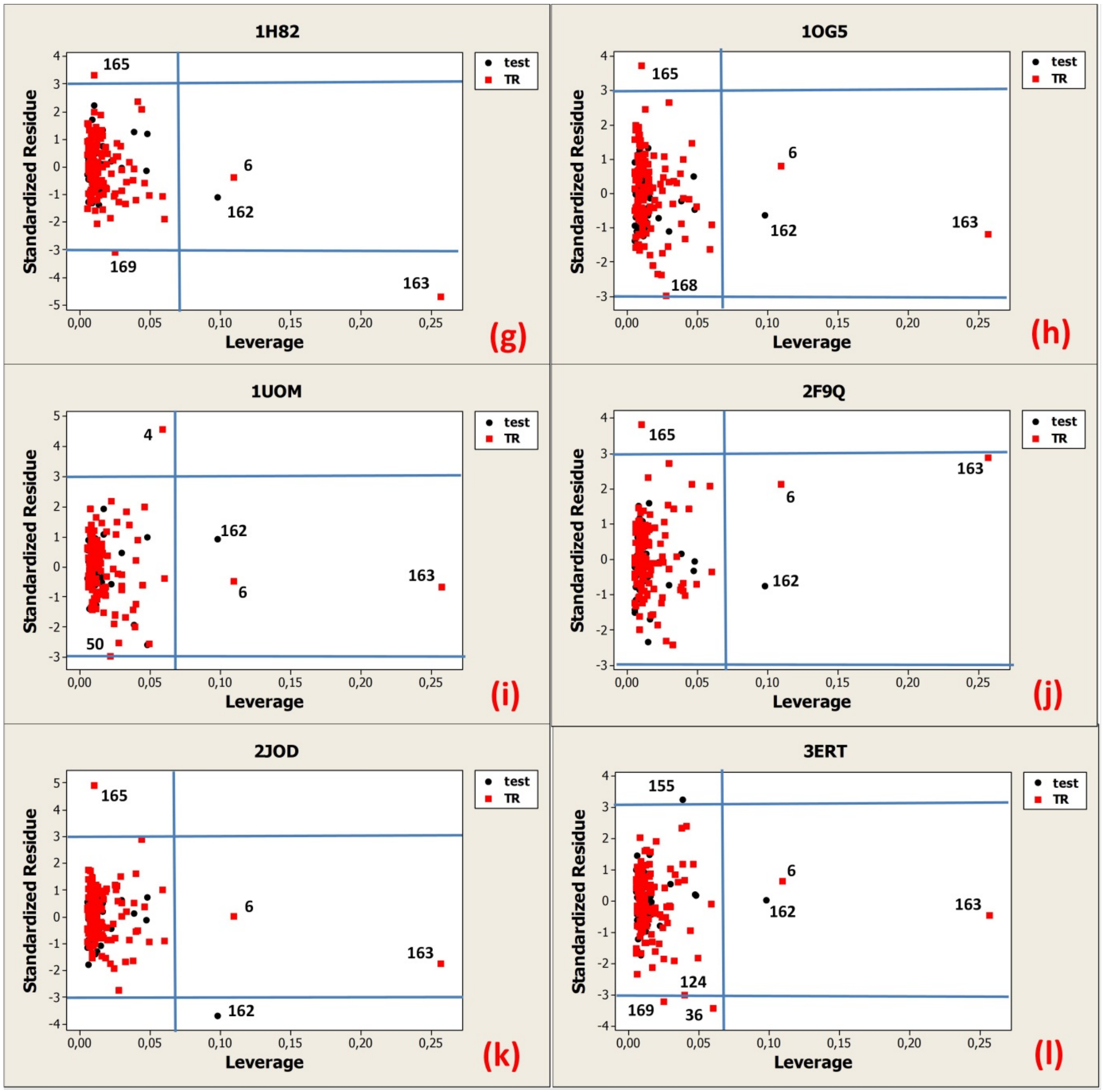

Figure S6. Williams plots: standardized residuals versus leverage for Model 1_regression based on QPpolrz and topological diameter (TD) for the following responses: binding scores for (g)- 1H82; (h)- 1OG5; (i)- 1UOM; (j)- 2F9Q; (k)- 2J0D; (l)- 3ERT.

Additionally, for better analysis we created Tables S7 and S8 in *SM1* section. In Table S7 we have considered FDs outside the warning leverage threshold (h*) as well as outside the square area between ±3 standard deviation units (σ) in Model 1_regression. Models for all responses were analyzed. The structures of FDs outside pointed limits were shown in Table S8.

Firstly, the FDs characterized by leverage (hat) value that exceeds the warning leverage threshold were considered. Thus, **FD6**, **FD162,** and **FD163** are the most influential on the structural domain of the models as they are located outside the warning leverage threshold. These three FDs belong to the highly active FDs. **FD163** (BScores=7885) contain 8 ammonium NH4+, **FD162** (BScores=7417) contain 6 ammonium NH4. **FD6** (BScores=6923) has the longest alkyl chain bounded to the C60 core.

Secondly, the FDs outside ±3σ were considered. We have found here the least active fullerenes without functional groups with different carbon atoms in the fullerene core. These are **FD50** (BScores=4224) (C_{70}), **FD168** (BScores=3938) (C_{60}) and **FD169** (BScores=4399) ($C_{80}H_2$).

FD4, **FD36**, **FD44, FD155**, and **FD165** appeared to be also outside ±3σ (see *Supplementary Material S1* Tables S7 and S8). **FD4** (Bscores=7257) belongs to the most active FD. It is attached to the C60 core with a cyclopropane 3-membered ring and contains two benzene rings and eight-CH_2-, two-COOH and two amide groups. **FD36** (BScores=6923) is connected to the C60 core with two benzene rings and containing two pyridine rings, two-NH_2, four -CH_3, and four ester groups. **FD44** (BScores=5673) belongs to the middle active. It is connected to the C60 core with a benzene ring and contains three-OH groups. High active **FD155** (BScores=6726) connected to the C60 core with pyrrolidine (5-membered ring) and containing aromatic nitrogen and three-CH_3. **FD165** (BScores=5975) contains: two phosphonate groups, four-CH_2-CH_3 and twelve hydroxyl groups -OH.

We can conclude that in most cases for the most responses the following FDs were outside ±3σ: the least active FDs without functional groups: **FD50** (pristine fullerene C_{70}); **FD 168** (pristine fullerene C_{60}) and **FD169** (fullerene $C_{80}H_2$). It should be highlighted that outside ±3σ was also located active **FD165** (BScores=5975) containing two phosphonate groups, four-CH2-CH3 and twelve hydrohyl groups -OH.

Table S7. FDs outside the limits: warning leverage threshold (h*) and outside the square area between ±3 standard deviation units (σ) in *Model 1_regression* related to responses: (a)-Average BScores; (b)-Binding affinity; binding scores for :(c)- 1D6U; (d)- 1E3K; (e)- 1GOS; (f)- 1GS4; (g)- 1H82; (h)- 1OG5; (i)- 1UOM; (j)- 2F9Q; (k)- 2J0D; (l)- 3ERT.

**Responses in** *Model 1_regression*	**FDs outside the warning leverage threshold (h*)**	**FDs outside the square area between ±3 standard deviation units (σ)**
(a)-Average BScores	FD6; FD162; FD163	FD50; FD169; FD165
(b)-Binding affinity	FD6; FD162; FD163	FD50; FD168; FD169; FD165
**Binding scores for**		
(c)- 1D6U	FD6; FD162; FD163	
(d)- 1E3K	FD6; FD162; FD163	FD165
(e)- 1GOS	FD6; FD162; FD163	
(f)- 1GS4	FD6; FD162; FD163	FD44
(g)- 1H82	FD6; FD162; FD163	FD169; FD165
(h)- 1OG5	FD6; FD162; FD163	FD168; FD165
(i)- 1UOM	FD6; FD162; FD163	FD4
(j)- 2F9Q	FD6; FD162; FD163	FD165
(k)- 2J0D	FD6; FD162; FD163	FD165
(l)- 3ERT	FD6; FD162; FD163	FD169; FD36; FD 155

Table S8. Structure of chemicals (FDs) outside the limits: warning leverage threshold (h*) and outside the square area between ±3 standard deviation units (σ) in *Model 1_regression*.

<table>
<tr><th>FDs outside h*</th><th>FDs outside±3 σ</th></tr>
<tr><td rowspan="2">High active:

FD6 (BScores=6922.5) has the longest alkyl chain with alkenyl group -C=C- and 1 -COOH, -NH-
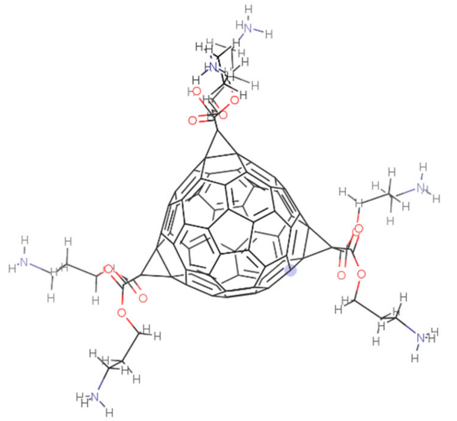
FD162 (BScores=7417,0) consists from 3 groups below with6NH3
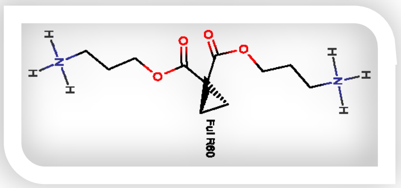
</td>
<td>Least active without functional groups
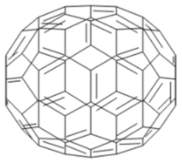
FD50(BScores=4224.3)-pristine fullerene C70
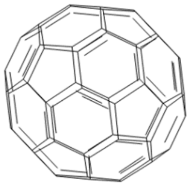
FD168-(BScores=3938.3)-pristine fullerene C60.

FD169 (BScores=4398.5) fullerene C80H2</td></tr>
<tr><td>The most active FDs
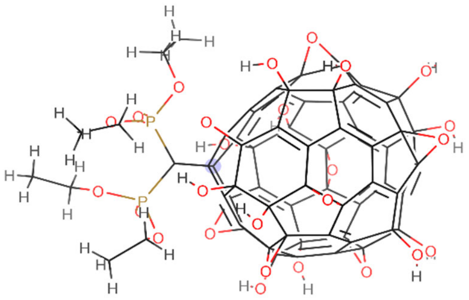
FD165(BScores=5975) contains: 2phosphonate groups 4-CH2-CH3; 12 hydrohyl groups -OH.

Group 2d (see in table SI 2)

Functional group see below:
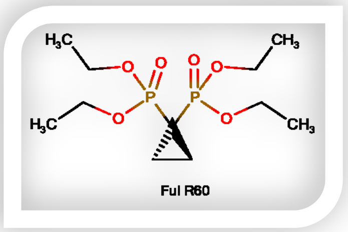
</td></tr>
</table>

<table>
<tr>
<td>
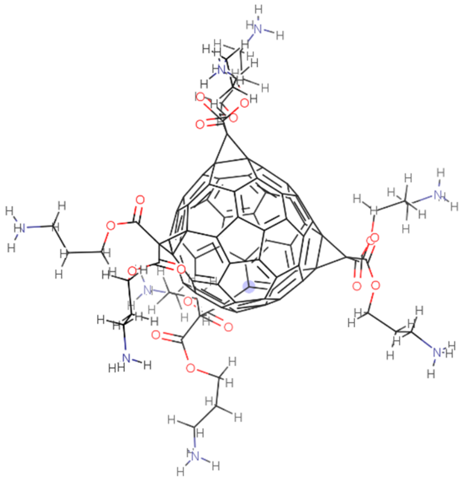
FD163(BScores=7885,2) consists of 4 groups below with8NH3
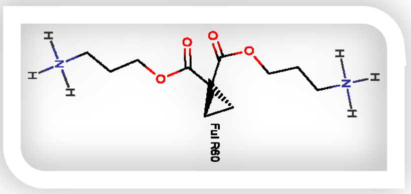

</td>
<td>
The most active FD4 (Bscores=7257) attached to the C60 core with cyclopropane 3-membered ring and containing two benzene rings and 8-CH2-, 2-COOH and 2amide groups
Group 5e (see in table SI 2)
Functional group see below:
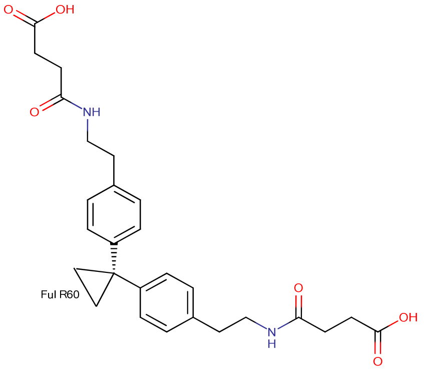

High active FD
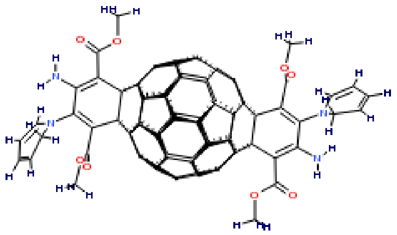
FD36(BScores=6922.5) connected to C60 core with 2 benzene rings and containing 2 pyridine rings, 2-NH2,4-CH3, 4 esther groups;
Group 5e (see in table SI 2).
See 2groups below:
Middle active
</td>
</tr>
</table>

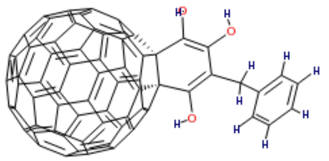

FD44 -(BScores=5673)connected to C60 core with benzene ring and containing 3-OH.
Group 5e (see in table SI 2)
Functional group see below:

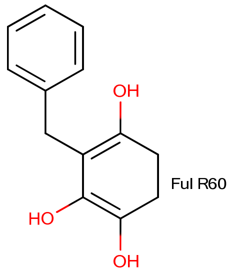

High active FD

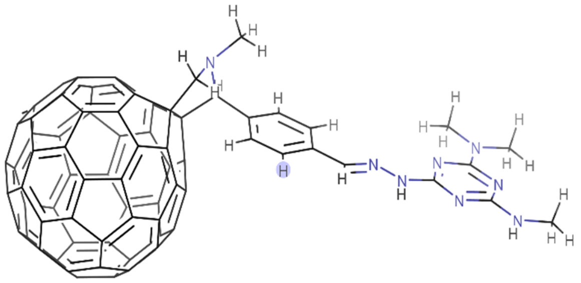

FD155--(BScores=6726) connected to to the C60 core with pyrrolidine (5-membered ring) and containing aromatic nitrogen and 3-CH3. Group 3a (see in table SI 2)
Functional group see below:

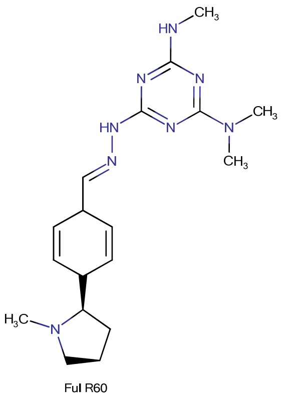

Model 2

In this part, we considered the Williams plots for Model 2_regression based on optimal Monte Carlo descriptors DCW. In this case standardized residuals were plotted versus leverage for the following responses: (a)-Average BScore; (b)-Binding affinity; (c)- 1D6U; (d)- 1E3K; (e)- 1GOS; (f)- 1GS4 (see Figure S7), while the Williams plots for the following responses:

Binding scores for (g)- 1H82; (h)- 1OG5; (i)- 1UOM; (j)- 2F9Q; (k)- 2J0D; (l)- 3ERT are represented in Figure S8.

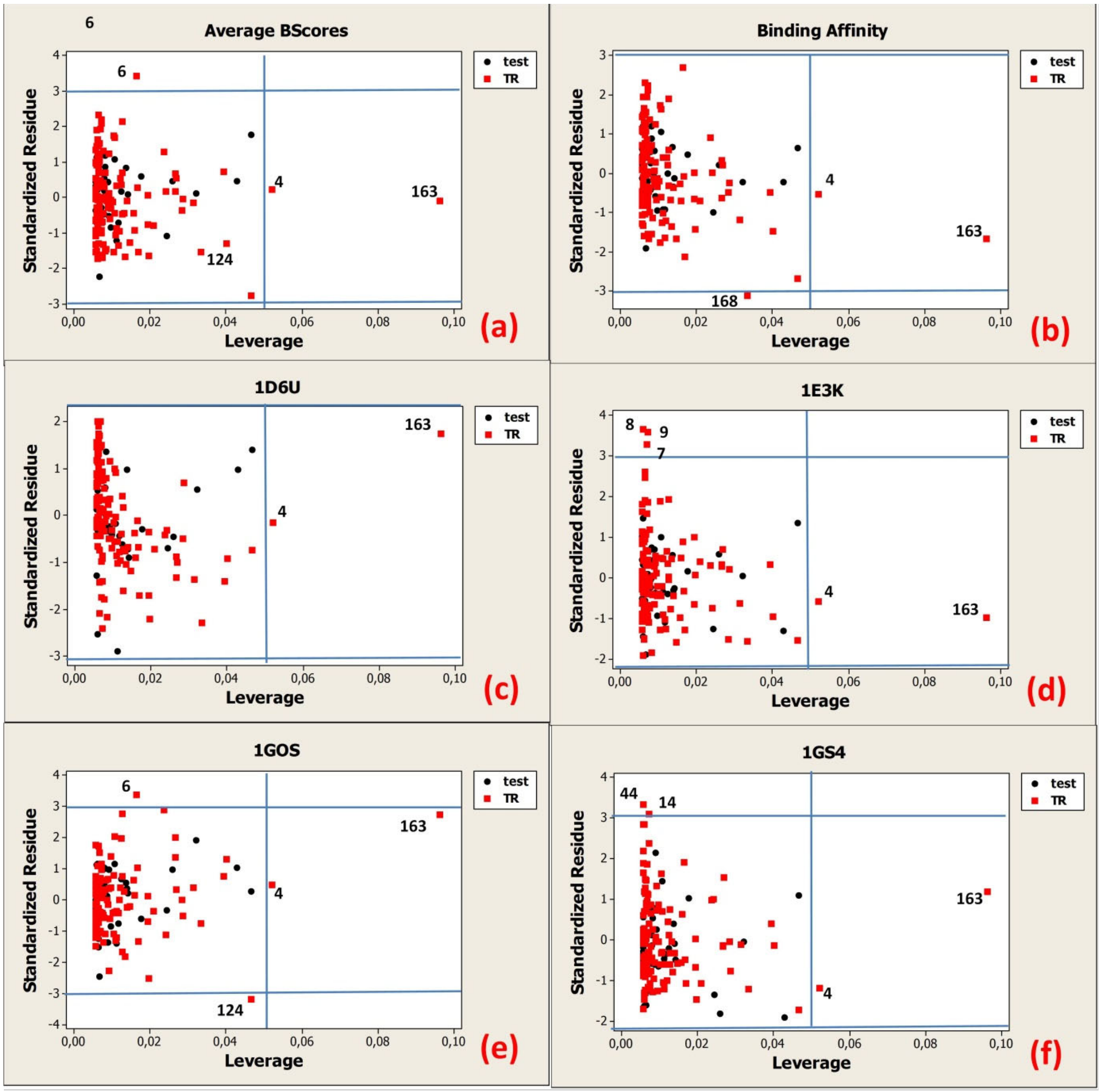

Figure S7. Williams plots: standardized residuals versus leverage for Model 2_regression based on the optimal Monte Carlo descriptors for the following responses: (a)-Average BScore; (b)-binding affinity; binding scores for (c)- 1D6U; (d)- 1E3K; (e)- 1GOS; (f)- 1GS4.

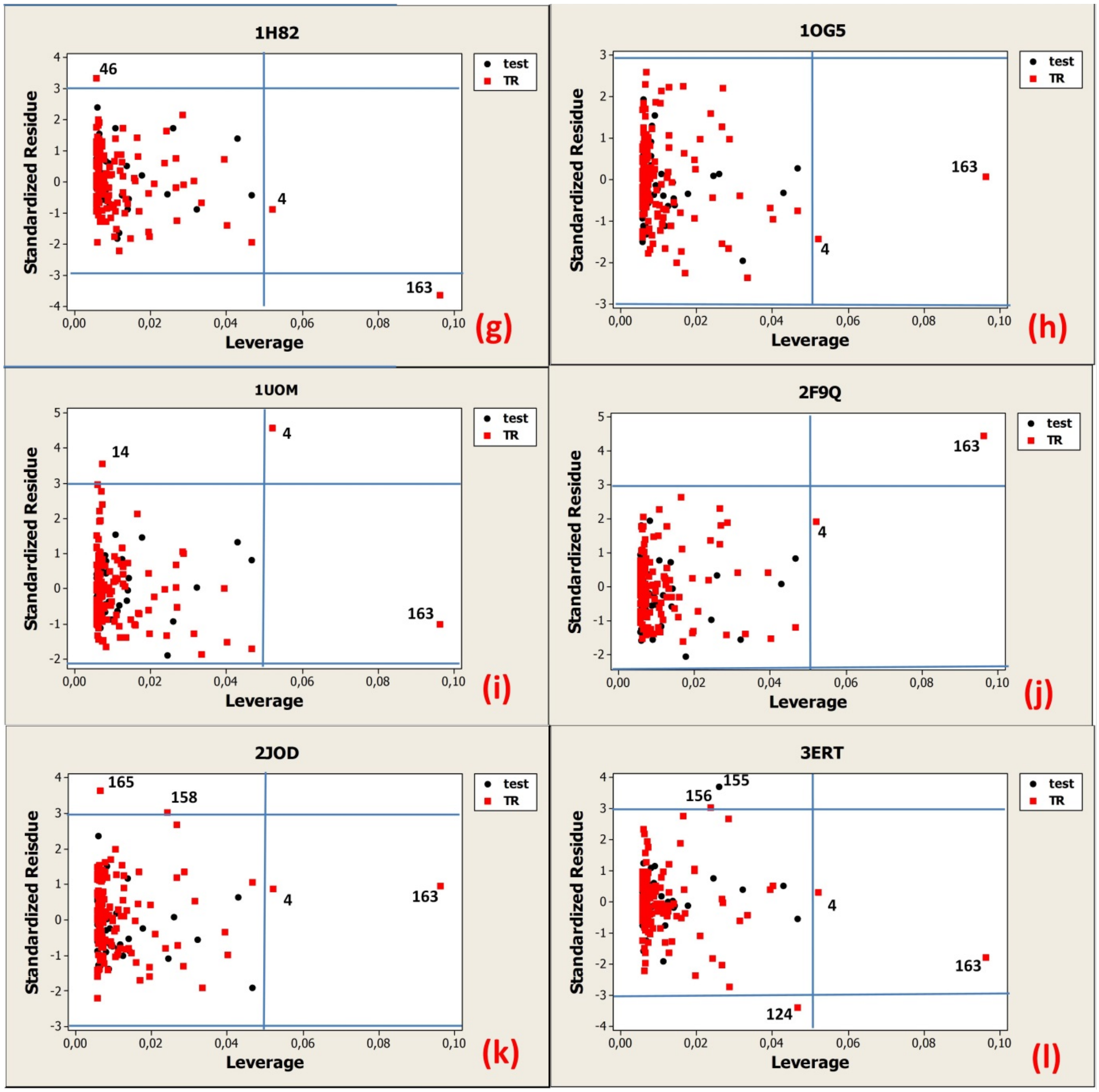

Figure S8. Williams plots: standardized residuals versus leverage for Model 2_regression based on the optimal Monte Carlo descriptors for the following responses: binding scores for: (g)- 1H82; (h)- 1OG5; (i)- 1UOM; (j)- 2F9Q; (k)- 2J0D; (l)- 3ERT.

Additionally, for analyzing FDs outside limits in Model 2_regression we created Tables S9 and S10 in *Supplementary Materials S1*.

See Table S9 with the indication of FDs outside the limits: warning leverage threshold (h*) and outside the square area between ±3 standard deviation units (σ) in Model 2_regression related to all responses.

Table S10 in the *Supplementary Materials S1* section corresponds to the structure of the chemicals (FDs) outside the limits for Model 2_regression.

Firstly, the FDs characterized by a leverage (hat) value that exceeds the threshold for warning leverage in Model 2_regression were examined. Thus, **FD4** and **FD163** are influential on the structural domain of the model as they are located outside the warning leverage threshold. **FD4** is a highly active compound with a long alkyl chain. **FD4** (BScores=7257) contains eight–CH_2–, two COOH, and two amide groups. **FD163** (BScores=7885) is also high active compound containing eight NH_3 (Table S10). Secondly, the FDs outside ±3σ were

considered. We have found diverse structures of FDs outside the limit of ±3 σ related to Model 2_regression (Table S10).

Table S9. FDs outside the limits: warning leverage threshold (h*) and outside the square area between ±3 standard deviation units (σ) in *Model 2_regression* related to responses: (a)-Average BScores; (b)-binding affinity; binding scores for: (c)- 1D6U; (d)- 1E3K; (e)- 1GOS; (f)- 1GS4; (g)- 1H82; (h)- 1OG5; (i)- 1UOM; (j)- 2F9Q; (k)- 2J0D; (l)- 3ERT.

**Responses in** *Model 2_regression*	**FDs outside the warning leverage threshold (h*)**	**FDs outside the square area between ±3 standard deviation units (σ)**
(a)-Average BScores	FD4; FD 163	FD6
(b)-Binding affinity	FD4; FD 163	FD168
**Binding scores for:**		
(c)- 1D6U	FD4; FD 163	
(d)- 1E3K	FD4; FD 163	FD7; FD8; FD9
(e)- 1GOS	FD4; FD 163	FD6; FD124
(f)- 1GS4	FD4; FD 163	FD14; FD44
(g)- 1H82	FD4; FD 163	FD46
(h)- 1OG5	FD4; FD 163	
(i)- 1UOM	FD4; FD 163	FD14
(j)- 2F9Q	FD4; FD 163	
(k)- 2J0D	FD4; FD 163	FD165; FD158
(l)- 3ERT	FD4; FD 163	FD124; FD155; FD156

Table S10. Structure of chemicals (FDs) outside the limits: warning leverage threshold (h*) and outside the square area between ±3 standard deviation units (σ) in *Model 2_regression*.

FDs outside h*	FDs outside±3 σ
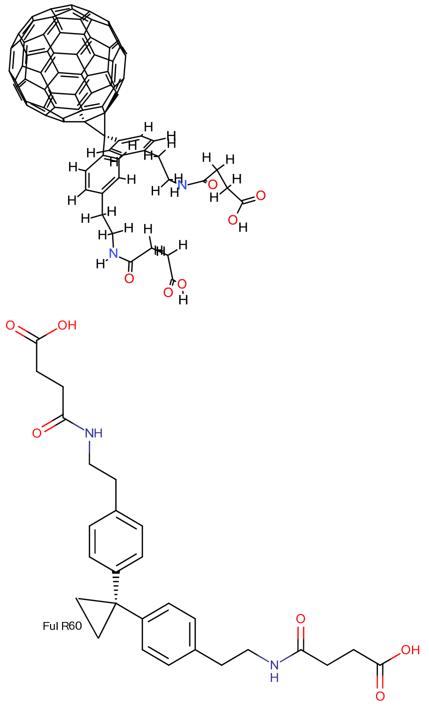 **FD4-**(Bscores= 7257) containe 8-CH2-; 2carboxyl-COOH; 2amide groups.	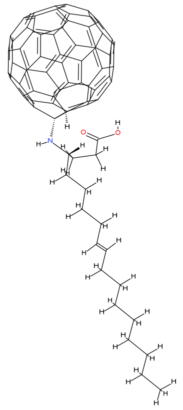 **FD6** (BScores=6923) has the longest alkyl chain with alkenyl group -C=C- and 1 -COOH, -NH- Functional group see below: 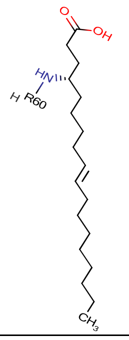

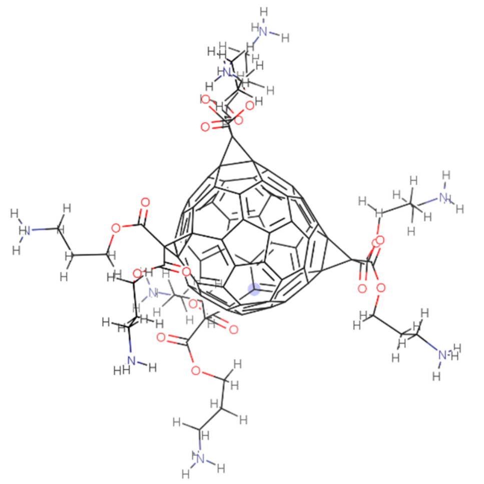

FD163(BScores=7885) consists of 4 groups below with 8NH3

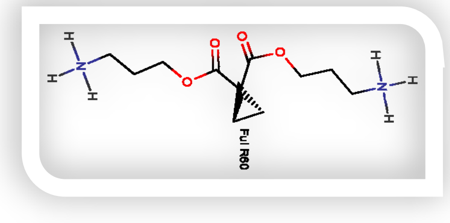

Group 1– FDs attached to C60 core with single bond through –NH- and containing alkyl groups (see in table SI 2)
Alkyl substituent

FD7 (BScores=5585)contains: 4-CH2-, 1>C(H)-, 1-NH2, 1-COOH.
Functional group see below:

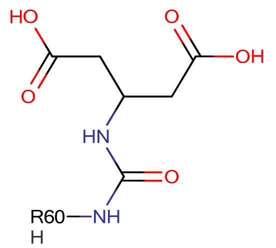

Group 1 (see in table SI 2)

FD8 (BScores=5710) contains: 1-COOH, 1-NH2, guanidine side chain.
Functional group see below:

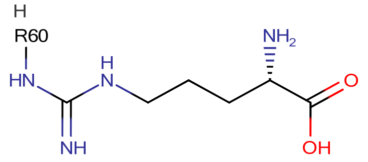

Group 1 (see in table SI 2)

FD9 (BScores=5591) contains: 2 amide groups, 2-COOH, 2-CH2-, 1>C(H)-
Functional group see below:

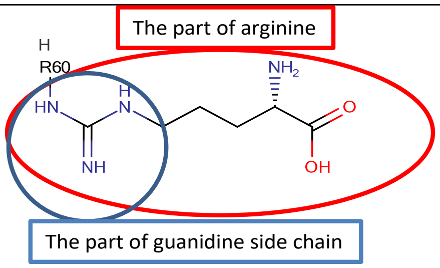

Group 1(see in table SI 2)

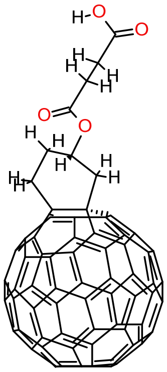

FD14 (BScores=5567) contains:ester group, -CH2-CH2-, 1-COOH

Functional group see below:

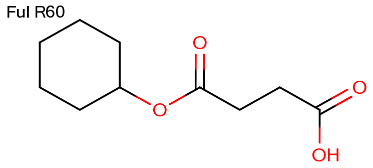

Group 4a FDs attached to the C60 core with 6-membered (cyclohexane) ring (see in table SI 2).

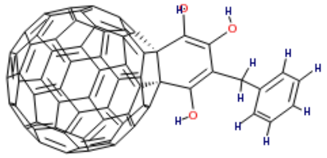

FD44 (BScores=5673) contains: benzene ring, 3-OH

Functional group see below:

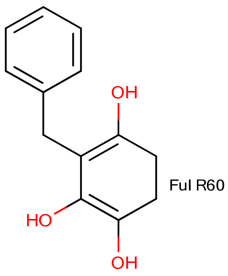

Group 5e– FDs connected to C60 core with benzene ring and containing pyridine groups and different kind of cycle groups (see in table SI 2)

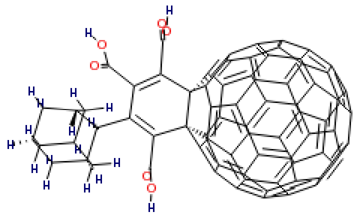

FD46 (BScores=5802) contains:bridged bicycle ring, 3-COOH.

Functional group see below:

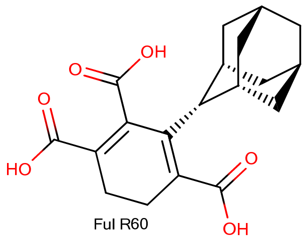

Group 5e– FDs connected to C60 core with benzene ring and containing pyridine groups and different kind of cycle groups (see in table SI 2)

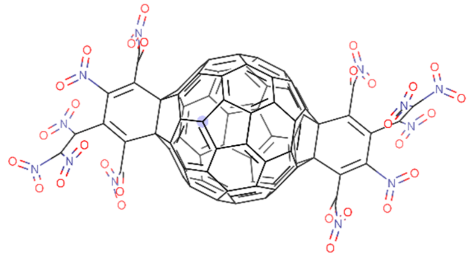

FD124 (BScores=6651) contains:2 groups: 12-NO2; 4 ketone groups

Functional group see below:

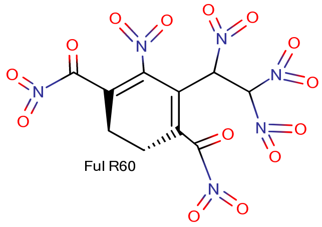

Group 5b1-the most active FDs connected to C60 core with benzene ring and containing 8-14 nitro groups –NO2(see in table SI 2)

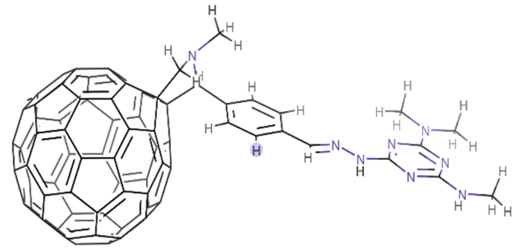

FD155 (BScores=6726) contains: 3-CH3.

Functional group see below:

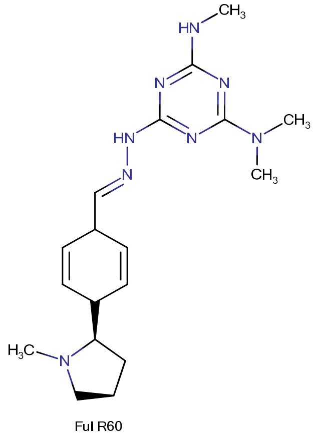

Group 3a-FDs attached to the C60 core with pyrrolidine (5-membered ring) and containing aromatic nitrogen (see in table SI 2)

FD156 (BScores=6802) contains: 4-CH3.

Functional group see below:

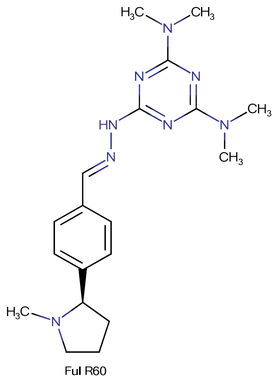

Group 3a-FDs attached to the C60 core with pyrrolidine (5-membered ring) and containing aromatic nitrogen (see in table SI 2).

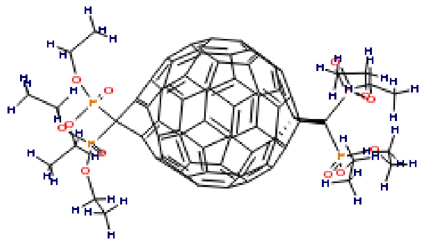

FD158 (BScores=6621) contains: 2 groups: 4phosphonate groups8-CH2-CH3

Functional group see below:

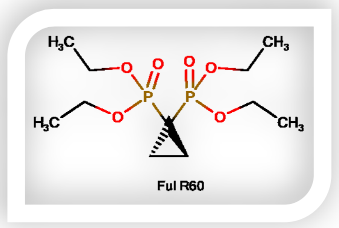

Group 2d

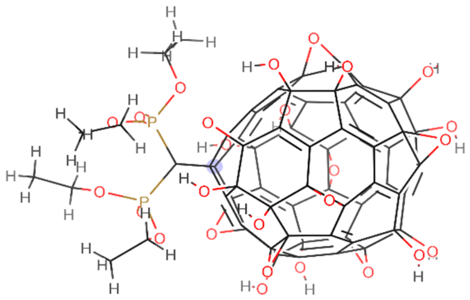

FD165 (BScores=5975) contains: 2phosphonate groups 4-CH2-CH3; 12 hydrohyl groups -OH.
Functional group see below:

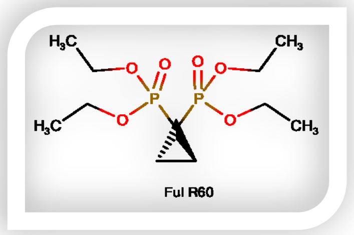

Group 2d

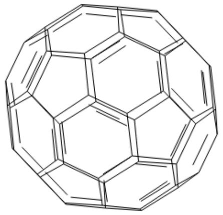

FD168(BScores=3938) Pristine fullerene C60.

CPANN models for prediction of binding activities using the descriptors QPpolrz and TD (CPANN model 1_) and the optimal descriptors DCW (CPANN model 2_)
Tables S11–S13.

Table S11. The statistical performance of *CPANN model 1* (M1) and *CPANN model 2*_(M2) related to training set.

Output variables	R^2_ M1_ Training	RMSE_ M1_ Training	R^2_ M2_ Training	RMSE_ M2_ Training
Average BScores (1)	0.9839	0.1264	0.9702	0.2324
Binding affinity (2)	0.9802	0.1402	0.9690	0.2288
Binding scores for 1D6U (3)	0.9121	0.2957	0.9011	0.7748
Binding scores for 1E3K (4)	0.9763	0.1534	0.9650	0.3252
Binding scores for 1GOS (5)	0.9840	0.1263	0.9567	0.3484
Binding scores for 1GS4 (6)	0.9584	0.2033	0.9444	0.4410
Binding scores for 1H82 (7)	0.9641	0.1887	0.9652	0.3334
Binding scores for 1OG5 (8)	0.9699	0.1730	0.9360	0.4571
Binding scores for 1UOM (9)	0.9691	0.1754	0.9389	0.4470
Binding scores for 2F9Q (10)	0.9842	0.1255	0.9478	0.3690
Binding scores for 2JOD (11)	0.9761	0.1543	0.9495	0.4570
Binding scores for 3ERT (12)	0.8925	0.3267	0.8392	0.4659

Table S12. The statistical performance of *CPANN model 1*(M1) and *CPANN model 2*(M2) related to test set.

Output variables	Q^2_ M1_test	RMSE_ M1_test	Q^2_ M2_test	RMSE_ M2_test
Average BScores (1)	0.9427	0.1722	0.9050	0.2995
Binding affinity (2)	0.9394	0.1756	0.9038	0.2879
Binding scores for 1D6U (3)	0.4205	0.3155	0.3754	0.7495
Binding sores for 1E3K (4)	0.8790	0.1868	0.7951	0.4302
Binding scores for 1GOS (5)	0.8702	0.2076	0.8470	0.3781
Binding scores for 1GS4 (6)	0.7758	0.2352	0.7406	0.4840
Binding scores for 1H82 (7)	0.8968	0.1862	0.8091	0.4442
Binding scores for 1OG5 (8)	0.7768	0.2523	0.8247	0.3885
Binding scores for 1UOM (9)	0.8077	0.2469	0.8195	0.3831
Binding Scores for 2F9Q (10)	0.8714	0.2279	0.8273	0.3752
Binding scores for 2JOD (11)	0.7752	0.2244	0.7985	0.4631
Binding scores for 3ERT (12)	0.8121	0.4008	0.5683	0.6763

Table S13. The statistical performance of *CPANN model 1*(M1) and *CPANN model 2*(M2) related to validation leave one out (LOO) procedure.

Output variables	Correlation coefficient Q^2cv_M1_LOO	RMSE_ M1_ LOO	Correlation coefficient Q^2cv_M2_LOO	RMSE_ M2_LOO
Average BScores (1)	0.9781	0.1475	0.9692	0.1752
Binding affinity (2)	0.9741	0.1605	0.9684	0.1774
Binding scores for 1D6U (3)	0.8346	0.4056	0.8619	0.3728
Binding scores for 1E3K (4)	0.9615	0.1957	0.9504	0.2224
Binding scores for 1GOS (5)	0.9704	0.1716	0.9538	0.2150
Binding scores for 1GS4 (6)	0.9460	0.2317	0.9310	0.2626
Binding scores for 1H82 (7)	0.9548	0.2120	0.9475	0.2286
Binding scores for 1OG5 (8)	0.9421	0.2400	0.9419	0.2405
Binding scores for 1UOM (9)	0.9532	0.2159	0.9367	0.2514
Binding scores for 2F9Q (10)	0.9643	0.1885	0.9477	0.2287
Binding scores for 2JOD (11)	0.9511	0.2205	0.9370	0.2506
Binding scores for 3ERT (12)	0.8899	0.3310	0.8544	0.3815

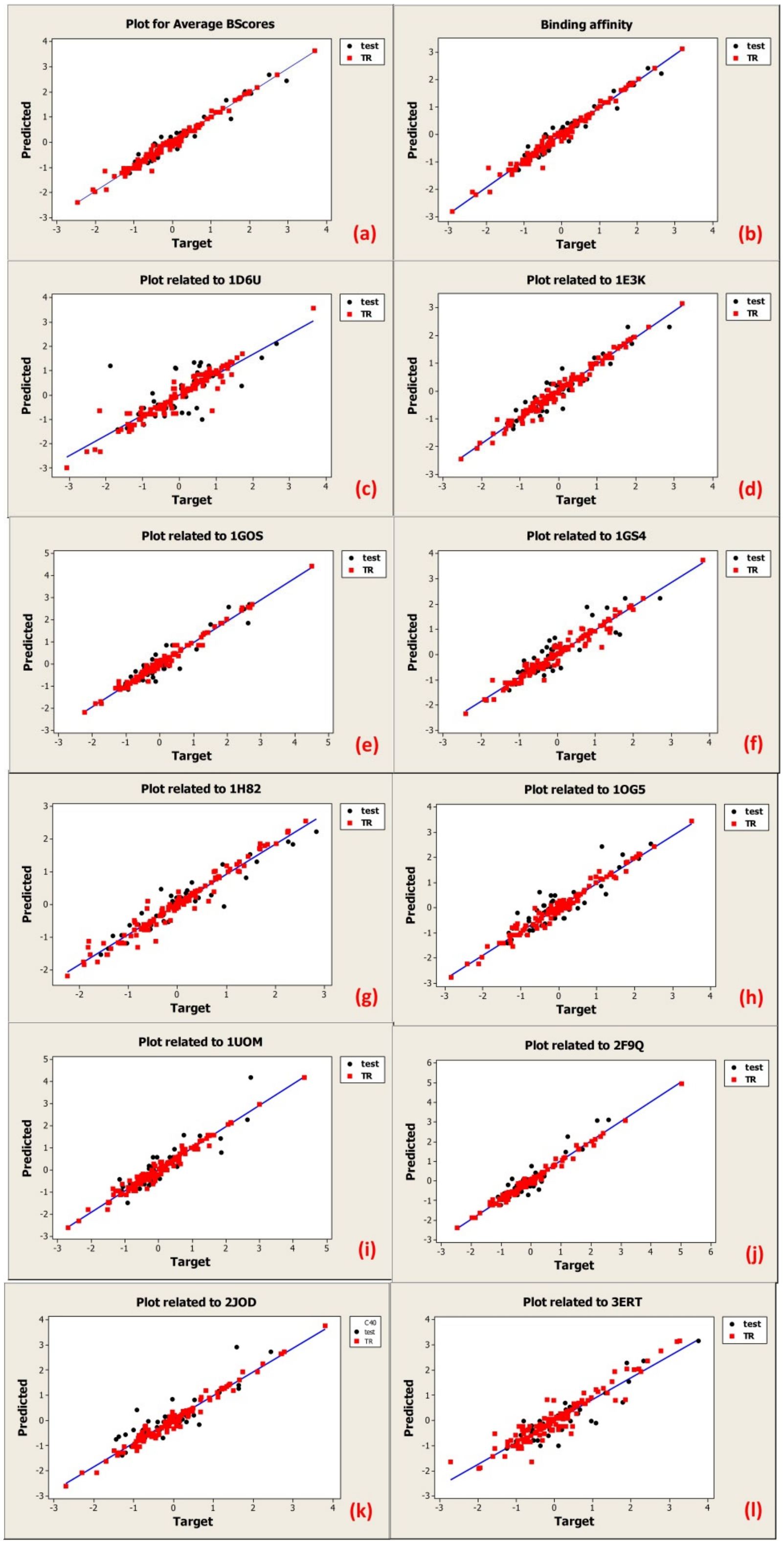

Figure S9. The plots of target response (binding affinity) vs. predicted one for *Model 1_CPANN*: for the following responses: (a)-Average BScore; (b)-binding affinity; binding scores for (c)- 1D6U; (d)- 1E3K; (e)- 1GOS; (f)- 1GS4; (g)- 1H82; (h)- 1OG5; (i)- 1UOM; (j)- 2F9Q; (k)- 2J0D; (l)- 3ERT.

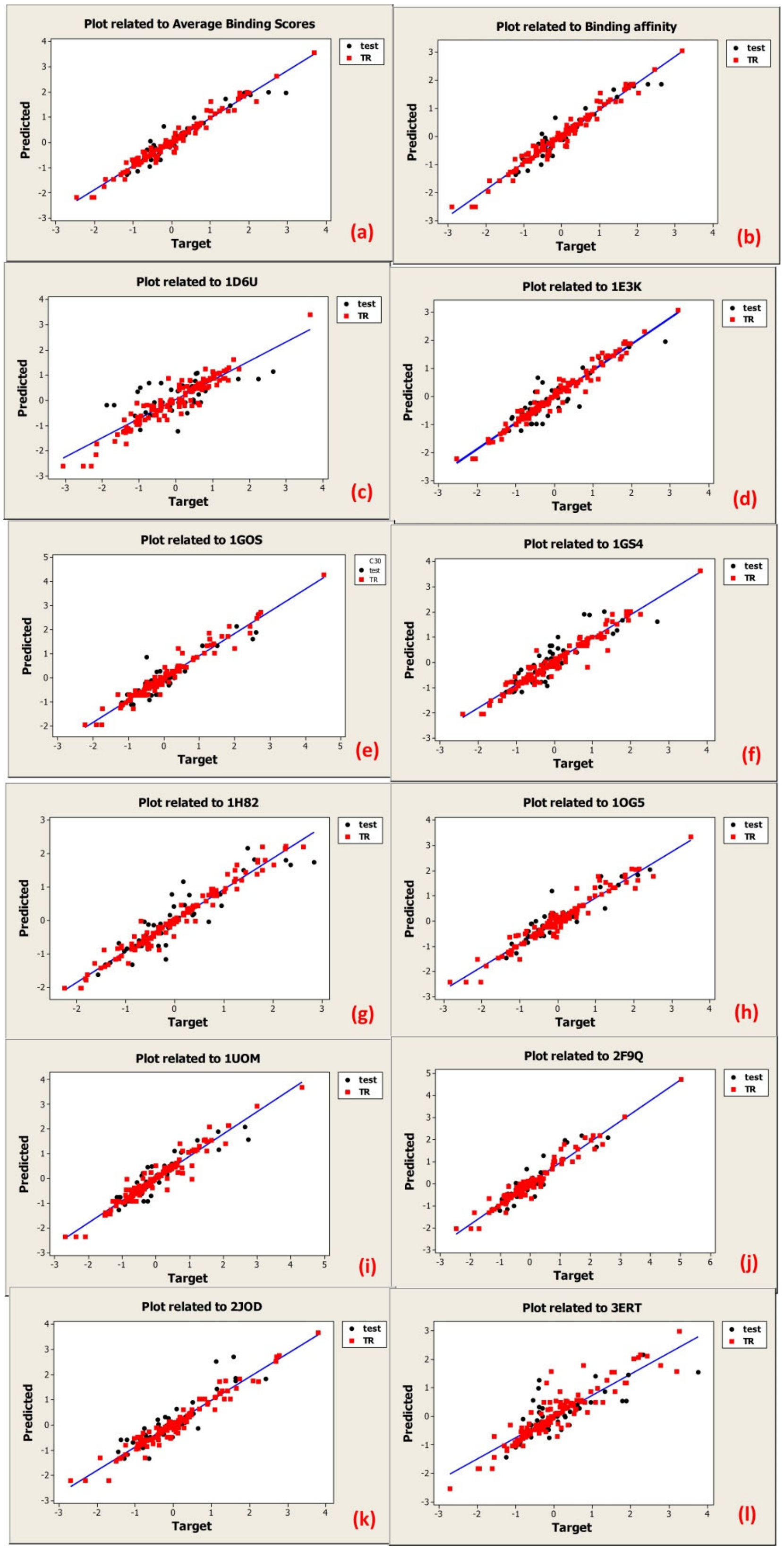

Figure S10. The plots of target response (binding affinity) vs. predicted one for *Model 2_CPANN*: for the following responses: (a)-Average BScore; (b)-binding affinity; binding scores for (c)- 1D6U; (d)- 1E3K; (e)- 1GOS; (f)- 1GS4; (g)- 1H82; (h)- 1OG5; (i)- 1UOM; (j)- 2F9Q; (k)- 2J0D; (l)- 3ERT.
