# Supplementary material for: Cheminformatics and Machine Learning Approaches to Assess Aquatic Toxicity Profiles of Fullerene Derivatives
Source: Int J Mol Sci. 2023 Sep 15;24(18):14160. doi: 10.3390/ijms241814160 (PMC10531479; doi:10.3390/ijms241814160)
Supplement: Supplementary file 1 [file ijms-24-14160-s001.zip › Supplementary Materials S2 (SMS2).pdf]

Cheminformatics and Machine Learning Approaches to Assess Aquatic Toxicity Profile of Fullerene Derivatives.

Natalja Fjodorova[a]*, Marjana Novič[a], Katja Venko[a], Bakhtiyor Rasulev[b], Melek Türker Saçan[c]; Gulcin Tugcu[d], Safiye Sağ Erdem[e], Alla P. Toropova[f], Andrey A. Toropov[f]

Supplementary Materials S2 (SMS2)

Table of content

Figure S11. ***Group1:*** FDs containing ***TA662-*** Aliphatic secondary and tertiary amines and ***TA665-*** Primary alkylamines: R = alkyl; molecules containing C, H and N atoms.
Figure S12. *Group 2*: FDs containing ***TA642-***Ammonium $NH3^+$ groups.
Figure S13. ***Group 3***: FDs containing 6-14 nitro groups -NO2 and the following TAs: ***TA628-*** β-Unsaturated nitro compounds, ***TA11521***- 4-nitrobenzene and ***TA667***- activated alkenes and alkynes.
Figure S14. *Group 3*: FDs containing 1-3 nitro groups -NO2 and the following TAs: ***TA628-*** β-Unsaturated nitro compounds and ***TA11521***- 4-nitrobenzene and ***TA667***- activated alkenes and alkynes.
Figure S15. *Group 4*: FDs containing phosphonate groups **- TA 617**- phosphoric acid ester.
Figure S16. *Group 5*: FDs containing halide groups (F) ***TA 634***, halogenated benzylic group ***TA 638*** and unsaturated nitro groups ***TA 628*** for FD 96 and 142.
Figure S17. *Group 6*: FDs containing nitrile groups with ***TA 626***-α, β-Unsaturated nitriles.
Figure S18. *Group* *7*: FDs containing ***TA 667***-activalted alkenes and alkynes and ***TA 631***-α, β-Unsaturated carboxylic acid.
Figure S19. *Group 8*: FDs containing alkyl amines ***TA 665.***
Figure S20. *Group 9*: FDs containing Carboxylic acid secondary amides ***TA 1181,*** Carboxylic acids ***TA 1176*** and Aliphatic alcohols ***TA 659.***
Figure S21. FD17 that has a high binding activity, but no structural alert for aquatic toxicity.

In the *SMS2* we represented diagrams with structures of considered FDs and structures of related to these FDs structural alerts TAs.

Figures in *SMS2* represented groups (1-9) of FDs dependent on structural alerts for aquatic toxicity with an indication of binding activity. For each FD we represented Binding scores activity as the value of Average Binding scores vs. values of Binding Scores for *Tc*AChE (BScores=value of AvBScores/value of BScores for *Tc*AChE).

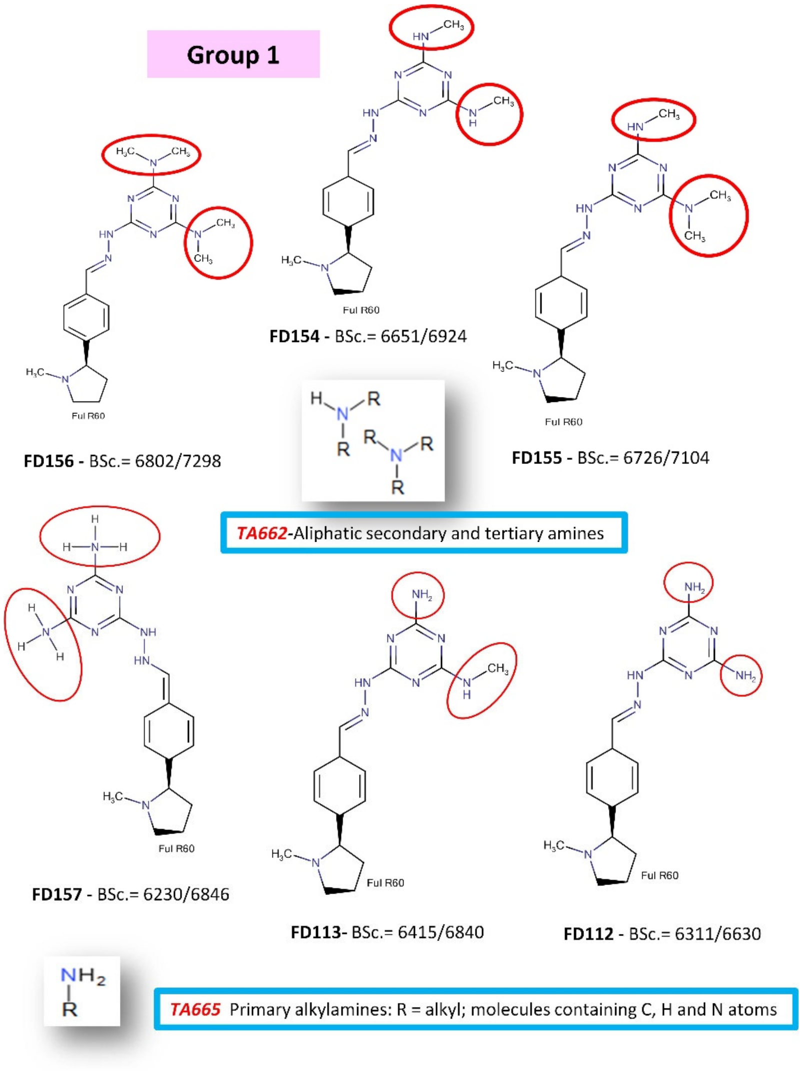

Figure S11. ***Group1:*** FDs containing ***TA662-*** Aliphatic secondary and tertiary amines and ***TA665-*** Primary alkylamines: R = alkyl; molecules containing C, H, and N atoms.

Figure S11 illustrated the structures of FDs containing structure alerts TA662- (Aliphatic secondary and tertiary amines) and TA665 (Primary alkyl amines). Some FDs (FD154, 155, 156) contain only TA662 while FD112 contains TA665. FD 113 contains both TA662 and 665.
This group 1 in the Kohonen top map in Figure 5a is located in the left upper corner and corresponds to the highest values of binding scores colored in red-orange-yellow. The highest binding scores for 1VOT was 7298 for FD 156 and 7104 for FD155 containing 2 TA662. FD112 containing 2 TA665 obtained the lower value of binding scores equal to 6630. We can suggest that FDs containing secondary and tertiary amine groups are more active than primary alkyl amine groups.
Considering the structures of this group of FDs we can't ignore that all these FDs contain pyrrolidine (5-membered ring) attached to the C60 core and all of these FDs contain aromatic nitrogen. We suppose that these groups can also benefit to binding activity and successively the toxicity of FDs.

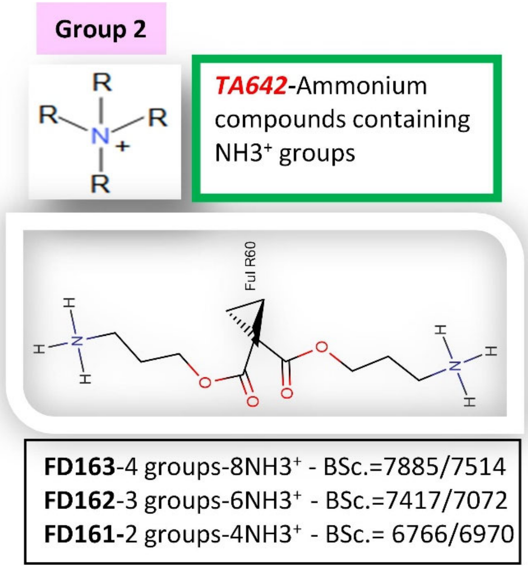

Figure S12. ***Group 2***: FDs containing ***TA642-***Ammonium NH3$^+$ groups.

Figure 12 illustrated the structures of FDs containing structure alert ***TA642*** (Ammonium compounds). This group contains the most active FDs. BScores for *Tc*AChE from 7514 to 6970. The number of NH3$^+$ groups counts from 4 (FD161) to 8 (FD163).

This *group 2* in the Kohonen top map in Figure 5a located in the left upper corner next to *group1*.

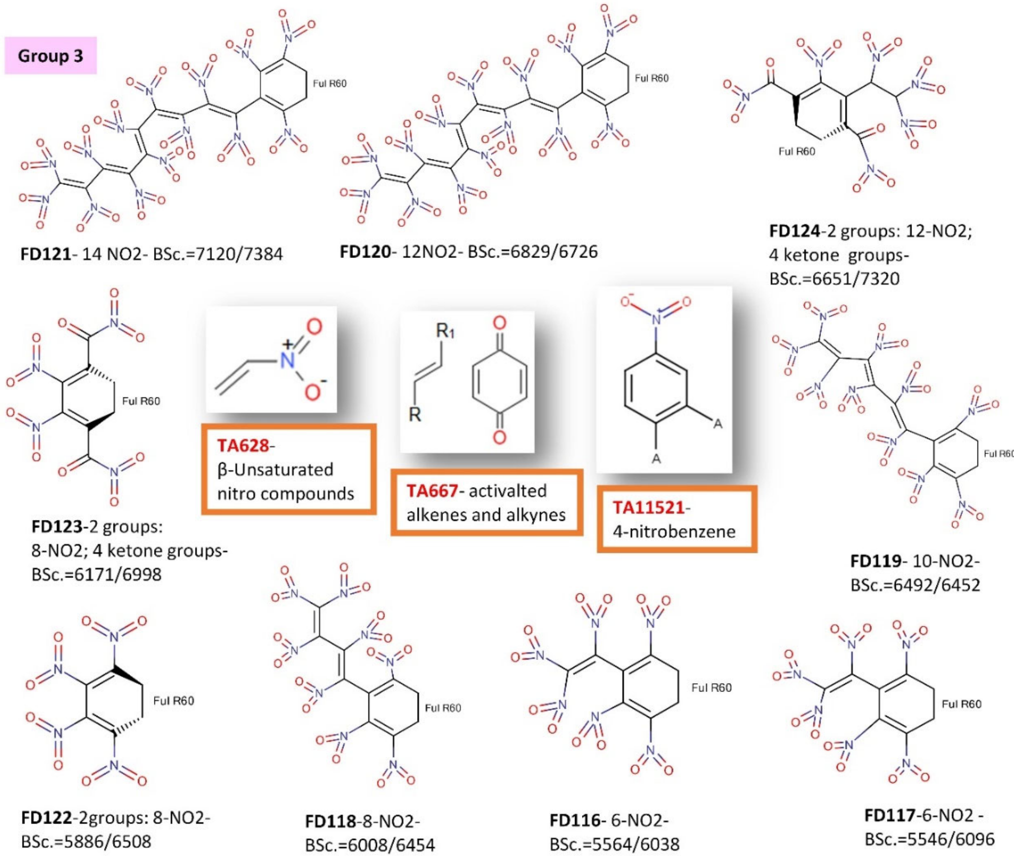

Figure S13. ***Group 3***: FDs containing 6-14 nitro groups -NO2 and the following TAs: ***TA628-*** β-Unsaturated nitro compounds, ***TA11521***- 4-nitrobenzene and ***TA667***- activalted alkenes and alkynes.

Group 3 contains also high active FDs. The activity depends mostly on the amount of –NO2 groups. Thus, the FD121 containing 14-NO2 have Av.BScores=7120 and Binding scores for 1VOT=7384 while FD117 containing 6-NO2 have Av.BScores=5546 and Binding scores for 1VOT=6096

Different structural alerts can sometimes overlaid each other.

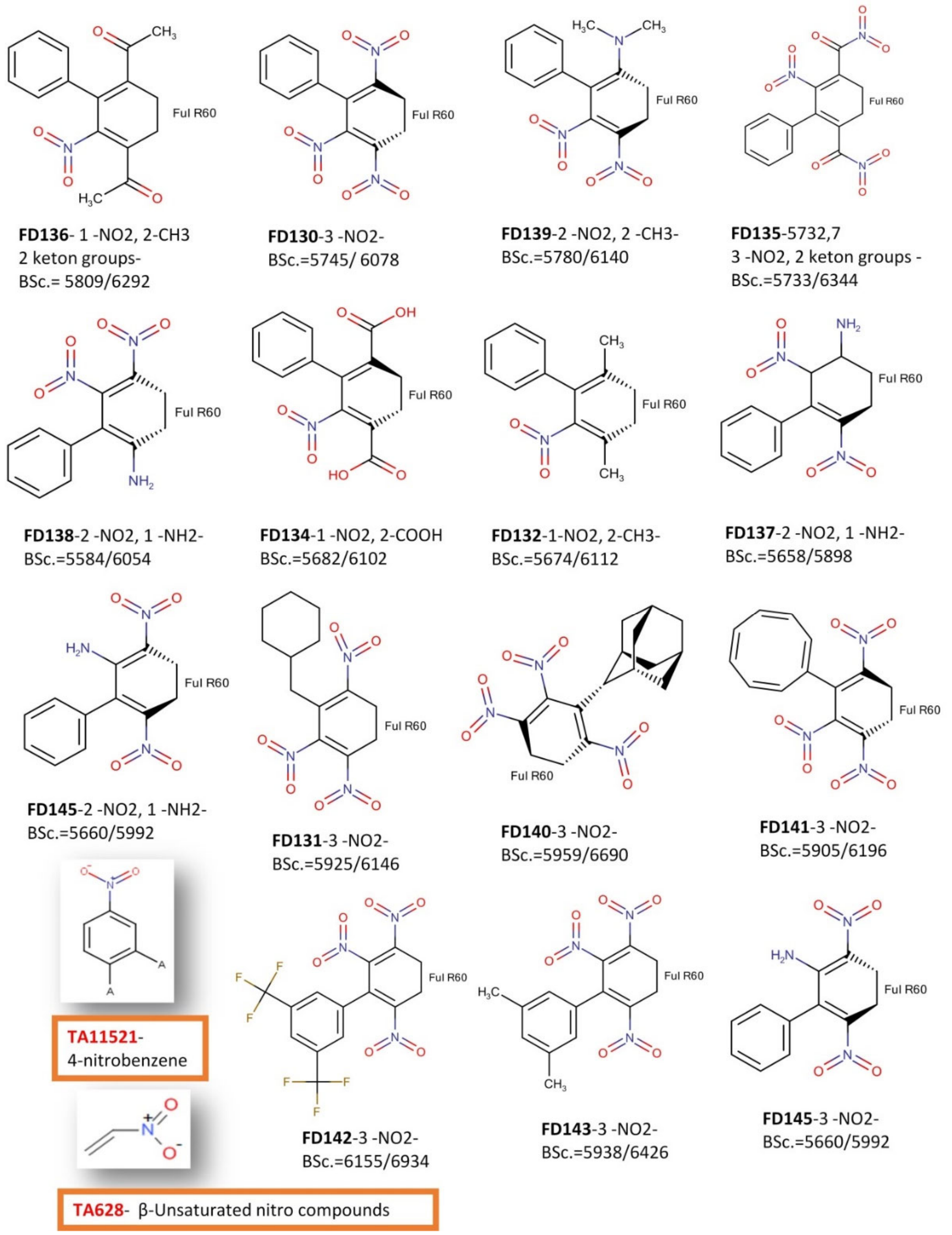

Figure S14. ***Group 3***: FDs containing 1-3 nitro groups -NO2 and the following TAs: ***TA628-*** β-Unsaturated nitro compounds and ***TA11521***- 4-nitrobenzene and ***TA667-*** activated alkenes and alkynes.

Group 3 in Figure S14 contains ***TA 628*** and ***TA11521*** and from 1 to 3 –NO2. **FD142** here contains also 6 –F groups. Therefore this is the most active FD BSc.=6155/6934. **FD140** contains besides of 3-NO2 also bridged bicycle ring (BSc.=5959/6690). The activities of others FDs in this group are from 5938 for av. BScores and 6426 for Binding scores for VOT1 (**FD143**) to BSc.=5584/6054 for **FD138**.

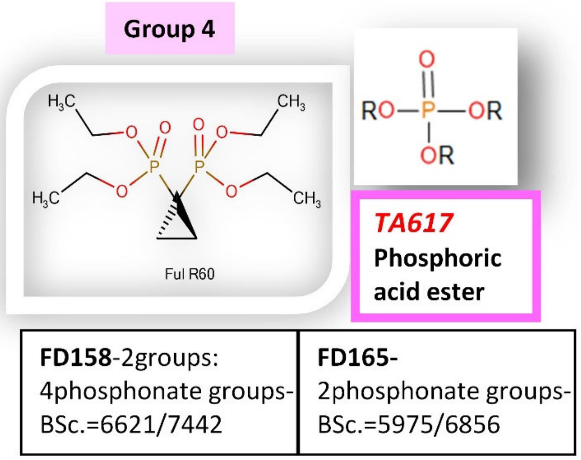

Figure S15. ***Group 4***: FDs containing phosphonate groups **- TA 617-** phosphoric acid ester.

Group 4 represents the FDs containing phosphonate groups. The more groups, the higher binding scores was obtained. Thus, **FD158-**containing 4phosphonate groups has BSc.=6621/7442 while **FD165** containing only 2phosphonate groups has BSc.=5975/6856.

In the paper by Tinkov et al., 2021 it was reported that for phosphate fragment the leading mechanism of toxic action is the inhibition of acetylcholinesterase (Čolović et al., 2013). It was experimentally proved on *Fathead minnow, Daphnia magna and Tetrahymena pyriformis.*

Tinkov et al., 2021- Tinkov, O., Polishchuk, P., Matveieva, M., Grigorev, V., Grigoreva, L., and Porozov, Y., 2020. The Influence of Structural Patterns on Acute Aquatic Toxicity of Organic Compounds. Mol. Inf. 39, 2000209 DOI: 10.1002/minf.202000209

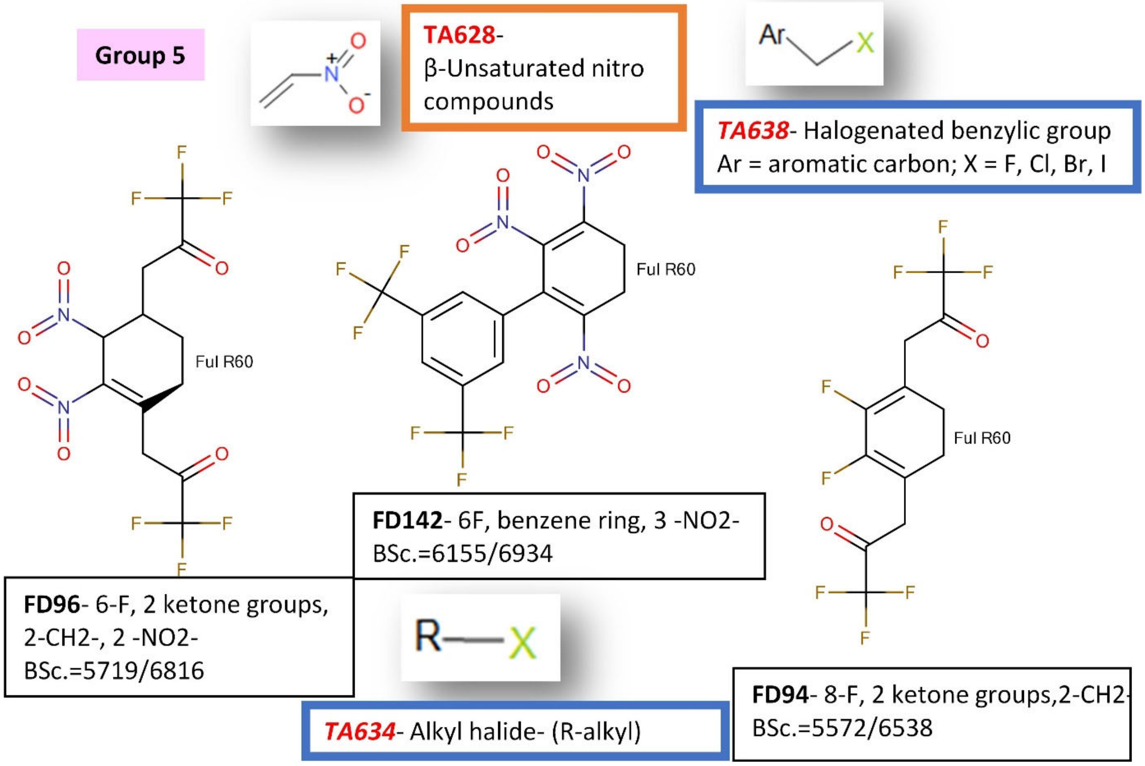

Figure S16. ***Group 5***: FDs containing halide groups (F) ***TA 634***, halogenated benzylic group ***TA 638*** and unsaturated nitro groups ***TA 628*** for FD 96 and 142.

Group 5 represents FDs containing halide groups (F) ***TA 634***, halogenated benzylic group ***TA 638*** and unsaturated nitro groups ***TA 628*** for **FD 96** and **FD142**. The binding scores for 1VOT (*Tc*AChE) here reached to 6934-6538.

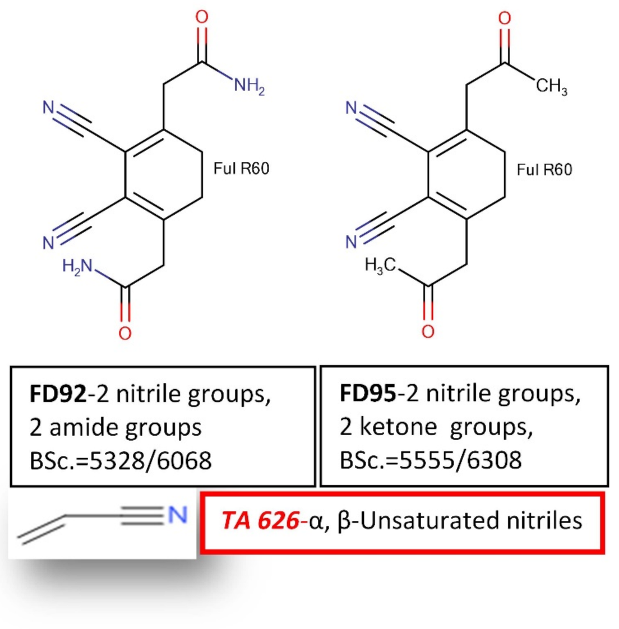

Figure S17. ***Group 6***: FDs containing nitrile groups with ***TA 626***-α, β-Unsaturated nitriles.

Unsaturated nitriles form group 6 with ***TA 626*** and binding scores for 1VOT (*Tc*AChE)=6068-6308.

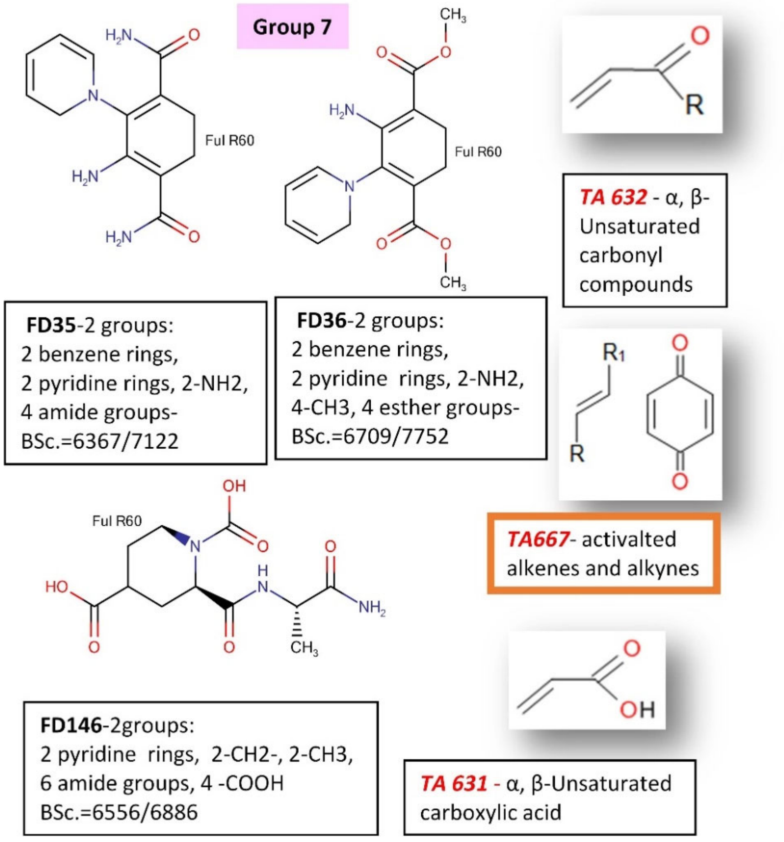

Figure S18. ***Group 7***: FDs containing ***TA 667-***activalted alkenes and alkynes and ***TA 631-***α, β-Unsaturated carboxylic acid.

Group 7 of active FDs (**FD35, 36** and **142**) was composed of FDs containing ***TA 631, TA 632*** and ***TA 667***.
Binding scores for 1VOT (*Tc*AChE)=7752-6886.

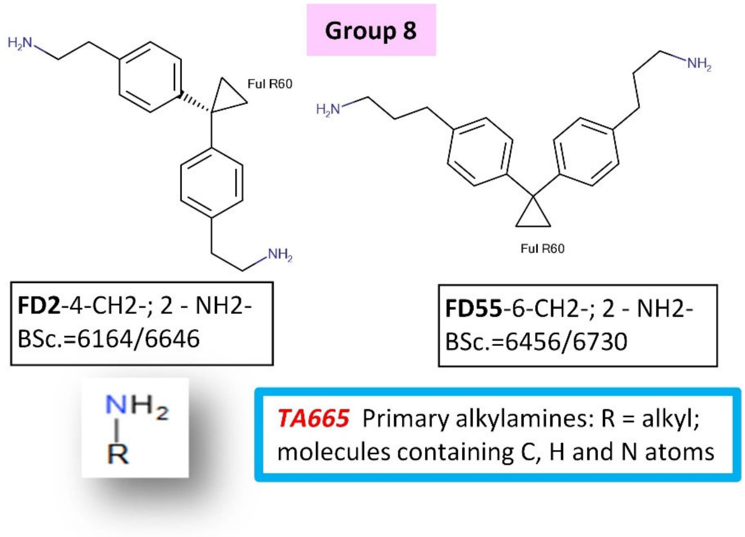

Figure S19. ***Group 8***: FDs containing alkyl amines ***TA 665.***

Group 8 (**FD2** and **FD55**) belong to the active FD located at the top right side of Kohonen map in Figure 5a. Binding scores for 1VOT (*Tc*AChE)=6730-6646.

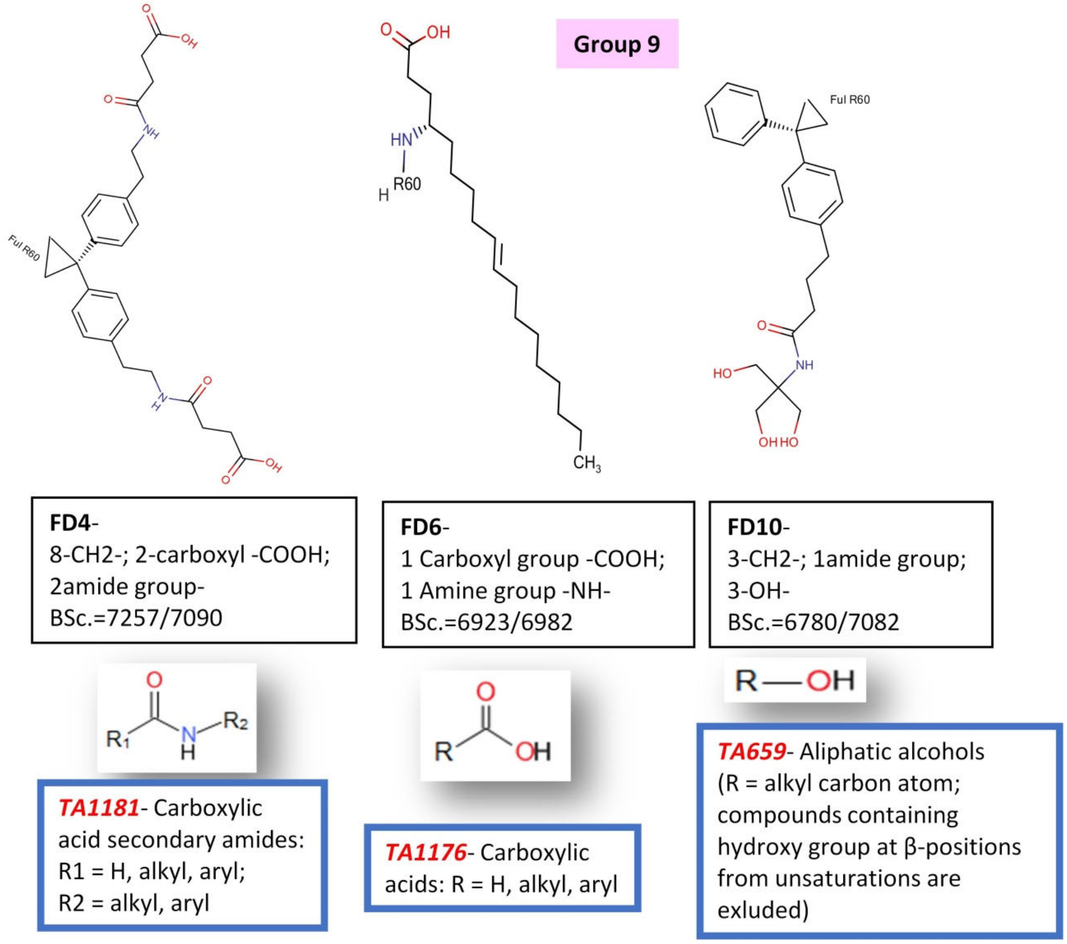

Figure S20. ***Group 9***: FDs containing Carboxylic acid secondary amides ***TA 1181,*** Carboxylic acids ***TA 1176*** and Aliphatic alcohols ***TA 659.***

Group 9 (**FD4, 6** and **FD55**) belong to the most active FD located at the top middle side of Kohonen map in Figure 5a (orange colour). Binding scores for 1VOT (*Tc*AChE)=7090-6982. They contain ***TA1181-*** Carboxylic acid secondary amides***, TA1176-*** Carboxylic acids and ***TA659*** Aliphatic alcohols**.**

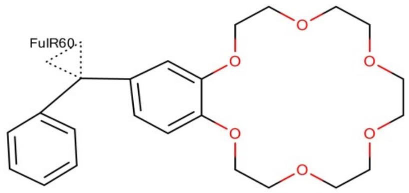

FD17-
1Crown-6 ether group
BSc.=6766/7328

Figure S21. FD17 that has a high binding activity, but we didn't find structural alert for aquatic toxicity.

FD17 has a high binding activity (BScores=7328), but we didn't find structural alert for aquatic toxicity. In Figure 5a **FD 17** located in upper part of top map in the middle close to group 9.

FD17 (see Figure S21) has complicated structure and SA for aquatic toxicity was not found for this compound. But because it has great value of binding scores we can suggest this FD to insert in the list for potential toxic compounds.
